# Involvement of β-defensin 130 (DEFB130) in the macrophage microbicidal mechanisms for killing *Plasmodium falciparum*

**DOI:** 10.1038/srep41772

**Published:** 2017-02-09

**Authors:** Mohamad Alaa Terkawi, Ryo Takano, Atsushi Furukawa, Fumi Murakoshi, Kentaro Kato

**Affiliations:** 1 National Research Center for Protozoan Diseases, Obihiro University of Agriculture and Veterinary Medicine, Inada-cho, Obihiro, Hokkaido 080-8555, Japan; 2 Frontier Research Center for Advanced Material and Life Science, Department of Orthopedic Surgery, School of Medicine, Hokkaido University, Kita 21, Nishi 11, Kita-ku, Sapporo, Hokkaido 001-0021, Japan; 3 Laboratory of Biomolecular Science, Faculty of Pharmaceutical Sciences, Hokkaido University, Kita-12, Nishi-6, Kita-ku, Sapporo 060-0812, Japan; 4 Research Center for Global Agromedicine, Obihiro University of Agriculture and Veterinary Medicine, Inada-cho, Obihiro, Hokkaido 080-8555, Japan

## Abstract

Understanding the molecular defense mechanism of macrophages and identifying their effector molecules against malarial parasites may provide important clues for the discovery of new therapies. To analyze the immunological responses of malarial parasite-induced macrophages, we used DNA microarray technology to examine the gene profile of differentiated macrophages phagocytizing *Plasmodium falciparum*-parasitized erythrocytes (iRBC). The transcriptional gene profile of macrophages in response to iRBCs represented 168 down-regulated genes, which were mainly involved in the cellular immune response, and 216 upregulated genes, which were involved in cellular proteolysis, growth, and adhesion. Importantly, the specific upregulation of β-defensin 130 (DEFB130) in these macrophages suggested a possible role for DEFB130 in malarial parasite elimination. Differentiated macrophages phagocytizing iRBCs exhibited an increase in intracellular DEFB130 levels and DEFB130 appeared to accumulate at the site of iRBC engulfment. Transfection of esiRNA-mediated knockdown of DEFB130 into macrophages resulted in a remarkable reduction in their antiplasmodial activity *in vitro*. Furthermore, DEFB130 synthetic peptide exhibited a modest toxic effect on *P. falciparum in vitro* and *P. yoelii in vivo*, unlike scrambled DEFB130 peptide, which showed no antiplasmodial activity. Together, these results suggest that DEFB130 might be one of the macrophage effector molecules for eliminating malarial parasites. Our data broaden our knowledge of the immunological response of macrophages to iRBCs and shed light on a new target for therapeutic intervention.

Malaria remains one of the top public health concerns worldwide, killing nearly 0.5 million people every year. Malaria is caused by apicomplexan protozoa of the genus *Plasmodium*, of which, *Plasmodium falciparum* causes the most deadly infection^1^. The emergence of resistance to the current antimalarial drugs has been increasingly documented in many endemic regions of the world. Therefore, a new frontline antimalarial therapy is urgently needed^2^. An understanding of a host protective mechanism that clears blood-stage malarial parasites should provide important information for the design of novel antimalarial medications^3^.

Macrophages play key roles in the innate immune response against infection through their ability to rapidly recognize, phagocytize, and kill microorganisms. Upon recognition by macrophages, foreign agents are internalized and engulfed into phagosomes. Then, the fused phagosomes mature into phagolysosomes and expose the engulfed microorganisms to various antimicrobial effectors^4^. Macrophages produce large numbers of proteases, hydrolytic enzymes, and antimicrobial peptides that are important defense components against bacteria, fungi and viruses^4^,^5^.

Recently, interest in antimicrobial host-defense peptides (AMPs) and their synthetic derivatives for the development of innovative therapeutic agents has gained momentum^5^. Antimicrobial peptide databases and their application against pathogenic microorganisms including malarial parasites are available at the APD website (http://aps.unmc.edu/AP/main.php). The potent antimicrobial activities of AMPs and their derivatives stem from their cationic charge, hydrophobicity, and amphipathicity. These attributes allow them to attach to and insert into membrane bilayers to form pores via barrel-stave, carpet, or toroidal-pore mechanisms. AMPs are classified into four major classes: β-sheet, α-helical, loop, and extended peptides^6^. Their preferential interaction with microbes rather than the mammalian host cells is most likely due to the microbial membrane composition that is rich in anionic phospholipids and lacks cholesterol^7^,^8^. Therefore, AMPs of macrophages that mediate the elimination of malarial parasites may offer a promising choice for the design of new and effective treatment compounds.

In the current study, we transcriptionally analyzed a gene profile of macrophages phagocytizing iRBC in an attempt to define the macrophage molecular defense mechanism against infection. This gene profile led us to identify DEFB130 as an antimicrobial host-defense peptide against malarial parasites.

## Results

### Response of differentiated macrophages to iRBCs

**Figure 1:**
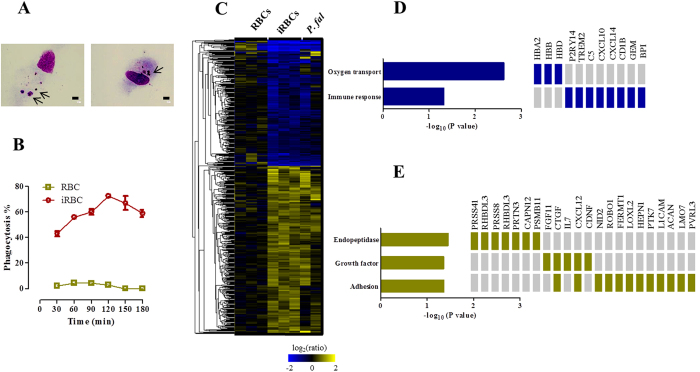
Response of human macrophages to iRBCs. (**A**) Parasitized erythrocytes or malaria pigment phagocytized by differentiated macrophages. Cells were cultured with enriched-iRBCs for 2 h on coverslips and then the coverslips were stained with Giemsa solution. Arrows indicate the phagocytized iRBCs and pigment. Scale bar = 10 μm. (**B**) Percentage of phagocytosis of iRBCs and RBCs at different time point of incubation. Each bar represents the mean ± SEM for each group (n = 3). (**C**) Gene profile of human differentiated macrophages cultured for 2 h with human non-infected RBCs (n = 3), enriched-iRBCs (n = 3), and saponin-treated iRBCs (*P. fal.*, n = 2) according to microarray analysis. (**D**) Clusters of down-regulated genes and (**E**) upregulated genes according to GO enrichment analyses.

Differentiated macrophages were cultured with iRBCs and their phagocytic activity was determined by counting the percentage of macrophages that engulfed iRBCs or malaria pigment (Fig. 1A) at different time points of incubation. The percentage of phagocytic macrophages gradually increased, peaked at 2 h post-culturing, and then decreased (Fig. 1A). In contrast, differentiated macrophages showed very low but detectable phagocytic activity to RBCs (Fig. 1B). These results suggest that differentiated macrophages specifically phagocytize iRBCs and may expose their effector molecules to the engulfed iRBCs in the early stage of phagocytosis. To gain insight into the immunological responses of macrophages phagocytizing iRBCs, we performed gene transcriptional analyses by using oligonucleotide-based DNA microarrays. Macrophages were cultured with RBCs, iRBCs or saponin-treated iRBCs for 2 h, and were then subjected to the microarray analyses. Among the 42,545 probes tested, 237 probes were significantly down-regulated and 310 probes were upregulated in macrophages phagocytizing iRBCs compared with those phagocytizing RBCs (Fig. 1C). Gene regulation in macrophages cultured with iRBCs appeared to be consistent with that in macrophages cultured with saponin-treated iRBCs, suggesting that the differential regulation of these genes was a result of macrophage activation induced by parasite-derived components (Fig. 1C). Functional annotation of these genes led us to identify 168 and 216 genes that were differentially down- and up-regulated, respectively, in the macrophages phagocytizing iRBCs (Supplementary Tables S1 and S2). Functional enrichment analyses according to Gene Ontology (GO) specifications were used to further determine the biological relevance of these genes. The down-regulated genes were involved in oxygen transport activity (*HBB*, *HBA2*, and *HBD*) and immune response (*P2RY14*, *TREM2*, *C5*, *CXCL10*, *CXCL14*, *CD1B*, *GEM*, and *BPI*), whereas the upregulated genes were cellular proteases (*PRSS41*, *RHBDL3*, *PRSS8*, *PRTN3*, *ADAM19*, *CAPN12*, and *PSMB11*), growth factors (*FGF11*, *CTGF*, *IL7*, *CXCL12*, and *CDNF*), or involved in adhesion (*NID2*, *ROBO1*, *FERMT1*, *LOXL2*, *HEPN1*, *PTK7*, *CTGF*, *L1CAM*, *CXCL12*, *ACAN*, *LMO7*, and *PVRL3*) (Fig. 1D,E). To further validate the transcriptional microarray results, we performed qRT-PCR for some of the upregulated genes, including *SMTNL2*, *DEFB130, RIT2*, *SYTL4*, *PRSS41*, *GRP*, and *ADAM19*. Notably, the qRT-PCR analysis data were highly consistent with the microarray analysis data (Table 1). The differential expression of the targeted genes was also confirmed by qRT-PCR in macrophages isolated from three different donors and cultured with iRBC (Table 1). Taken together, macrophages induce a unique gene profile in a response to iRBCs that reflects their potency for combating malarial infection.

### DEFB130 is a macrophage effector molecule against *Plasmodium falciparum*

**Figure 2:**
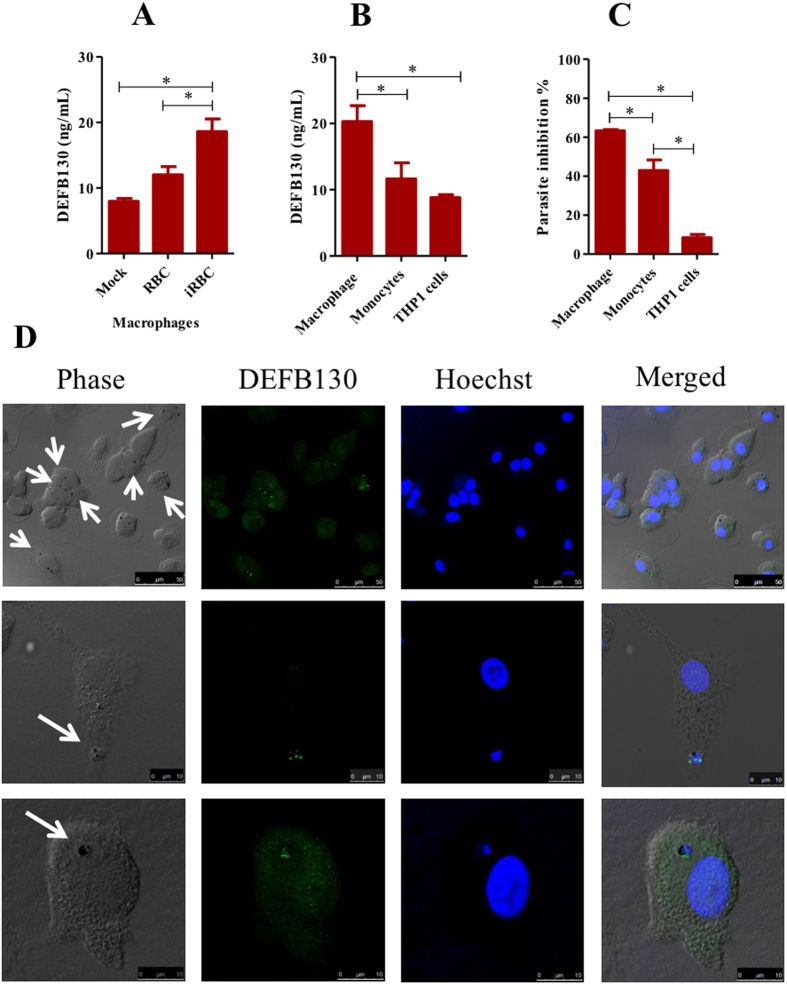
Analysis of the DEFB130 response in macrophage-phagocytizing iRBCs. (**A**) Protein level of DEFB130 in macrophages cultured with iRBCs or RBCs as detected by ELISA. Differentiated macrophages were either cultured alone with medium (Mock) or cultured with RBC and iRBCs for 2 h in 96-well plates. Human RBCs or iRBCs were added to macrophage cultures at 1:20 ratio. (**B**) Comparison of DEFB130 production from different phagocytic cells. The level of protein was quantified by ELISA in differentiated macrophages, primary monocytes, and THP1 cells cultured with iRBCs. (**C**) Parasite inhibition after culturing phagocytes with enriched-iRBCs. Percentage of inhibition was calculated based on the parasite biomass in the wells containing iRBC with no phagocytic cells. Phagocytic cells were cultured with enriched iRBCs (1:20) for 2 h in 96-well plates. Each bar represents the mean ± SEM for each group (n = 5) and the results are representative of at least two independent experiments. *Indicates a significant difference between the groups as analyzed by one-way ANOVA analysis of variance, followed by Tukey’s multiple-comparison test. (**D**) Intracellular localization of DEFB130 in macrophages phagocytizing iRBCs examined by confocal microscopy. Differentiated macrophages cultured with iRBCs for 2 h on coverslips and then the coverslips were fixed and stained by specific antibody. Green indicates specific reaction with DEFB130 antibody and blue indicates Hoechst nuclear staining. Arrows indicate the phagocytized iRBCs. Scale bar is indicated on each image.

**Figure 3:**
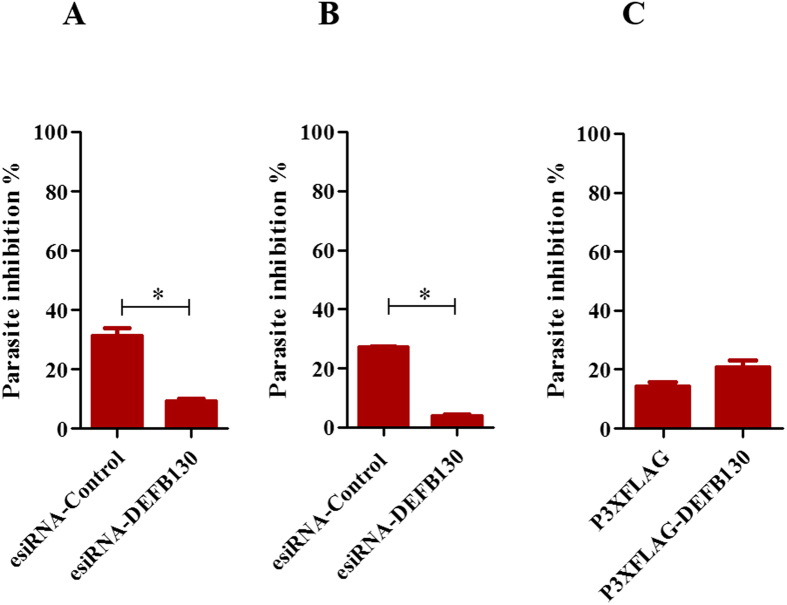
Antiplasmodial activity of genetically manipulated macrophages *in vitro*. (**A**) Parasite inhibition after culturing control esiRNA- or DEFB130-esiRNA-transfected macrophages with enriched iRBCs for 2 h. Macrophage were harvested 48 h after transfection and co-cultured with enriched iRBCs for 2 h. Thereafter, cells were lysed by 2 cycles of freezing and thawing and their supernatants were examined by using an ELISA kit for parasite biomass. (**B**) Growth inhibition assay after subculturing of iRBCs from macrophages co-cultures with fresh human RBCs for 24 h. The iRBCs cultured with either control esiRNA- or DEFB130-esiRNA-transfected macrophages were suspended and 10 μl were transferred into new 96-well plate, and mixed with 90 μl fresh RBCs at hematocrit of 2%. Cultures were incubated for 24 h and the parasite biomass was determined by ELISA. (**C**) Parasite inhibition after culturing DEFB130-overexpressing THP1 cells with enriched iRBCs for 2 h. Percentage of inhibition was calculated based on the parasite biomass in the wells containing iRBC with no phagocytic cells. Phagocytic cells were cultured with iRBC (1:20) for 2 h in 96-well plates. Each bar represents the mean ± SEM for each group (n = 5) and the results are representative of at least two independent experiments. *Indicates a significant difference between the groups as analyzed by Student’s t test.

The microarray results identified β-defensin 130 (*DEFB130*) as one of the top three upregulated genes in macrophages phagocytizing iRBCs. To validate this finding at the protein expression level, we quantified DEFB130 protein in an ELISA. Consistent with the microarray and qRT-PCR results, we found that macrophages cultured with iRBCs exhibited higher production of DEFB130 than macrophages cultured with RBC (Fig. 2A). Interestingly, the differentiated macrophages exhibited a significantly higher level of DEFB130 than that exhibited by monocytes or the monocyte-derived cell line THP1 in response to iRBC (Fig. 2B). The higher level of DEFB130 coincided with an increase in the growth inhibition of *P. falciparum in vitro* (Fig. 2C). Likewise, confocal laser scanning microscopic examination showed that macrophages phagocytizing iRBC had higher levels of DEFB130, which appeared within the cytosol of the macrophages and accumulated around the engulfed iRBC or malaria pigment (Fig. 2D). To verify the involvement of DEFB130 in the macrophage defense mechanisms against the parasites, DEFB130-knockdown and -overexpressing cells were prepared and the effects of manipulated cells were evaluated against the *in vitro* growth of parasites. Levels of DEFB130 were detected in transfected cell cultures by ELISA to confirm the efficacy of transfection (Supplementary Fig. S1). Strikingly, cultures of iRBCs with macrophages transfected with DEFB130-esiRNA exhibited significantly higher parasites biomass than these with macrophage transfected with control esiRNA (Fig. 3A). To further confirm this finding, co-cultured iRBCs with transfected macrophages for 2 h were subcultured with fresh human erythrocytes and their growth was determined after 24 h of culture. Parasitic biomass in cultures derived from DEFB130-knockdown macrophages was significantly higher than that in control cultures (Fig. 3B). Moreover, the exogenous addition of DEFB130 to THP1 cells slightly enhanced the toxic activity of THP1 cells against iRBC, but there was no statistically significant difference compared to that of vector-transfected cells (Fig. 3C). These data suggest that DEFB130 might be one of the effector molecules of macrophage for eliminating malarial parasites.

Given the importance of the cationic and amphipathic properties of β-defensins in their antimicrobial activity, we examined the structural features of DEFB130 and compared them with those of other defensins. DEFB130 is 7-kDa cationic and amphipathic peptide with a +9 net charge; it comprises six invariant cysteine residues and abundant lysine and arginine residues. CD spectroscopy analyses revealed that the spectrum of bacterial recombinant DEFB130 was characterized by a small negative band at 205 nm and a broad negative band at about 205–230 nm (Supplementary Fig. S2A). This same pattern was observed with synthetic DEFB130 peptide (data not shown). These results indicate that DEFB130 has an α-helix and β-sheet conformation and is stabilized by three disulfide bonds, with a folding pattern similar to other β-defensins. Interestingly, homology modeling of DEFB130 revealed the presence of a unique acidic region on the surface of the protein, which was not found in other β-defensins (Supplementary Fig. S2B–E). These data emphasize the antimicrobial properties of DEFB130 and its conservation among the β-defensins.

### DEFB130 synthetic peptide suppresses the growth of malarial parasites

**Figure 4:**
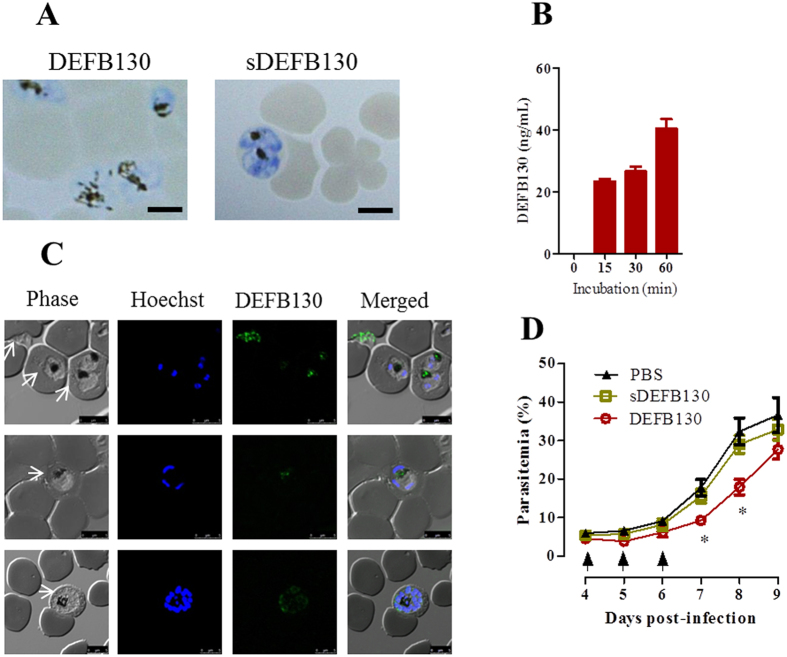
Antiplasmodial activity of DEFB130. (**A**) Morphological changes in the parasites after 3 h of culture in the presence of 50 μM peptide. Blood smears were stained with Giemsa stain. Scale bar = 10 μm. (**B**) Detection of DEFB130 in iRBCs by ELISA at different time points of incubation. Each bar represents the mean ± SEM for each group (n = 4) and the results are from individual experiment and are representative of two independent experiments. (**C**) Detection of DEFB130 in iRBCs after a 30-min treatment. Extracellular and late-ring stage parasites (upper panels), trophozoite (middle panels) and schizont (lower panels). Fixed blood smears were stained with specific antibodies and examined by confocal microscopy. Arrows indicate the parasites. Scale bar = 5 μm. Green indicates specific reaction with DEFB130 antibody and blue indicates Hoechst nuclear staining. (**D**) Parasitemia of C57/BL6 mice after treatment with DEFB130 peptide. Mice were infected with *P. yoelii* 17X-NL and on days 4, 5, and 6 p.i. were intravenously injected with DEFB130, sDEFB130, or PBS. Arrows indicate the time of injection. Parasitemia was monitored daily and on days 7 and 8 p.i. significant differences were observed. Each bar represents the mean ± SEM for each group (n = 5). *Indicates a significant difference between the groups as analyzed by one-way ANOVA.

To gain direct evidence of the antimicrobial effect of DEFB130 on malarial parasites, we examined the effect of synthetic DEFB130 peptide and its scrambled peptide on the *in vitro* growth of *P. falciparum*. Interestingly, DEFB130 peptide yielded IC_50_ values in the range of 43–49 μM, whereas its scrambled peptide had no apparent toxicity against *P. falciparum* parasites (Table 2). No hemolytic activity for DEFB130 was observed at concentrations up to 200 μM (data not shown). To examine the cytolytic activity of the peptide on the intracellular parasite, we incubated iRBCs with 50 μM DEFB130 for 3 h, and then stained blood smears with Giemsa and analyzed them by light microscopy. Remarkably, iRBCs treated with DEFB130 exhibited diffused hemozoin crystals within the cytoplasm of the parasites (Fig. 4A). Concurrently, the treated iRBCs were assayed by use of an ELISA and immunofluorescence, which confirmed the presence of DEFB130 within the iRBCs (Fig. 4B,C). To further assess the antimalarial activity of DEFB130 *in vivo*, mice were infected with *P. yoelii* 17NXL strain and then treated with synthetic peptides of DEFB130 or sDEFB130 on days 4, 5, and 6 post-infection. Consistent with the *in vitro* data, mice treated with DEFB130, but not those treated with the scrambled peptide, showed significantly reduced parasitemia on days 7 and 8 post-infection (Fig. 4D). The parasitemia of these mice was not significantly different on later days of the infection relative to the control mice (data not shown). These data suggest that DEFB130 has the pharmacological characteristics of a potential antimalarial compound. To precisely define the effective region of DEFB130, we synthesized analog peptides for the N-terminal (32 amino acids) and C-terminal (15 amino acids) domains of DEFB130 and examined their inhibitory effects against *P. falciparum* parasites *in vitro*. Strikingly, the analog peptide of the N-terminal but not the C-terminal domain was effective against the growth of parasites with IC_50_ values in the range of 90–93 μM (Table 2). Together, these results suggest that the maximal cytolytic activity of DEFB130 likely depends on the full cationic charge, hydrophobicity, and amphipathicity of the peptide.

## Discussion

Identifying host defense peptides that contribute to the control of malarial infection should aid in the design of novel therapeutic interventions. To this end, we studied the molecular defense responses of macrophages phagocytizing iRBCs or RBCs by means of transcriptional gene profile analyses. Macrophages phagocytizing iRBC showed a novel gene profile that was characterized by the down-regulation of their immune responses and the upregulation of a number of genes involved in cellular proteolysis, growth, and adhesion. This down-regulation of the immune response is most probably due to the pigment of parasites released after RBC lysis. Indeed, hemozoin has a negative effect on the function of macrophages, typified by a reduction in their phagocytic activity as well as their production of pro-inflammatory cytokines and the oxidative burst^9^. In contrast, the upregulation of protease gene expression in the macrophages may suggest a role for these enzymes in the destruction of iRBC. The modulation of macrophage adhesion in response to iRBCs is probably to facilitate and enhance iRBCs binding^10^. Our results support the concept that phagocytizing iRBCs and malarial pigment leads to altered physiological functions and biological properties of phagocytes^9^,^11^.

DEFB130 was one of the top upregulated genes in macrophages phagocytizing iRBCs. Importantly, this gene was not previously reported to be differentially regulated in macrophages in response to bacterial, viral, or parasitic pathogens^12^,^13^,^14^,^15^,^16^. Differentiated macrophages phagocytizing iRBCs have a high intracellular level of DEFB130 that accumulated at the site of the engulfed iRBCs. Our interpretation for the elevation of DEFB130 in macrophage phagocytizing iRBCs is that macrophage may utilize DEFB130 as a cysteine-rich cationic low molecular weight antimicrobial peptide to initiate intracellular parasite death within phagolysosome. Low molecular weight proteins can penetrate RBC membrane and accumulate to their target organelle of parasites leading to irreversible damage and death^3^. Consistently, our data showed that knockdown of DEFB130 expression in macrophages resulted in a reduced antiplasmodial activity *in vitro*. Furthermore, DEFB130 synthetic peptide had modest toxic effect against malarial parasites without affecting human RBCs at the micromolar level. Although the concentration of DEFB130 needed to effectively inhibit parasite growth *in vitro* appeared to be greater than that in the macrophages, the antiplasmodial effects of DEFB130 cannot be ignored; especially considering that the maximal antimicrobial effects of defensins likely occur in phagolysosomes^8^,^17^. Treating the mice with DEFB130 synthetic peptide resulted in a temporal decrease in parasitemia, which can be explained by the number of drug administration. Our further study is to examine the effects of prolonged administration of DEFB130 on the malarial parasites in mice.

The potent activity of β-defensin members is believed to be dependent on their cationic charge, hydrophobicity, and amphipathicity^8^. In support of this concept, neither scrambled DEFB130 peptide, which has the same amino acid composition and net positive charge but lacks the structure of the original peptide, nor the C-terminal domain of the DEFB130 peptide, which has a net positive charge of +1, was toxic to the parasites. Moreover, the N-terminal peptide of DEFB130 exhibited at least 2-fold lesser antiplasmodial activity than full length peptide. We therefore speculate that the amphipathic characteristics and net positive charge of DEFB130 may allow electrostatic interactions with the negatively charged phospholipid bilayers of the parasites inducing membrane permeabilization followed by leakage of the cellular content and cell death. In contrast, the lack of DEFB130 toxicity to host cells is likely due to the fact that the membrane of host cells is rich in cholesterol and has a low anionic charge that puts it out of the target range of defensins^6^,^7^,^8^. Another possibility for the selective effect of DEFB130 on iRBCs may be that infection with *P. falciparum* alters the permeability, fluidity, and protein and lipid composition of the RBC membrane, which may increase the binding affinity and insertion efficiency of the peptide to the membranes of the iRBCs relative to those of RBCs^18^.

DEFB130 has a high degree of conservation with DEFB1 and DEFB3, which have been previously shown to have toxic activities against various pathogens^19^,^20^. For example, at concentrations of 12.5–25 μM, DEFB1 and DEFB3 exert strong antibacterial effects against *Pseudomonas aeruginosa*, *Enterococcus faecalis*, and *Escherichia coli*^19^. Likewise, DEFB1 and DEFB3 have strong antifungal activities at concentrations of 2.5–7 μM^21^. Moreover, DEFB1-deficient mice are more susceptible to infection with *Candida albicans* and exhibit elevated systemic fungal burdens compared with wild-type mice^22^. DEFB3 inhibits human immunodeficiency virus type 1 replication and virion infectivity and at 50 μM completely inhibits herpes simplex virus type 1 replication^23^,^19^. Recombinant DEFB1 exhibits antitrypanosomal activities at 25 μM against the bloodstream form of *Trypanosoma brucei*^24^. The variation in the inhibitory doses of these β-defensins against these pathogens can be explained by the fact that their killing capacity is dependent on the ionic concentration in the medium^25^. An increase in the ionic strength of the medium with opposing charges of the cationic polypeptides can diminish their mutual attraction to anionic microbial surfaces^26^. These findings may explain why DEFB130 is toxic to malarial parasites only at high concentrations. Although the IC_50_ value of DEFB130 was relatively high, treatment with an effective dose resulted in morphological observations characterized by residual hemozoin cytosols within the cytoplasm of the parasites. This finding is consistent with reports on other antimicrobial peptides, such as NK2 and platelet factor 4, which have similar effects on malarial parasites^3^,^27^. Together, these data may suggest a conserved mode of action for these components. However, the mechanism by which platelet factor 4 kills malaria parasites is dependent on the ability of the peptide to lyse the digestive vacuole membrane of the parasites, leading to the dispersion of hemozoin throughout the parasites^3^. Our future studies will address the antiplasmodial mechanism of DEFB130 and involve the design of pharmacological analogs with higher potency toward malarial parasites.

In closing, the present study adds to our knowledge of the early response of macrophages to *P. falciparum-*parasitized erythrocytes and demonstrates for the first time the direct antiplasmodial activity of DEFB130. The molecular mechanism by which macrophages kill malarial parasites seems to be complex, and DEFB130 is likely one of their effector molecules for elimination of parasites. Continuing research to discover new antimicrobial components of host cells and their mode of action may aid in the design of novel antimalarial medications.

## Methods

### Ethics statement

Our research protocols for the use of human blood and animal experiments were approved by the Research Ethics Review Committee of the Obihiro University of Agriculture and Veterinary Medicine (approval numbers 2014–01 and 26–130, respectively), and informed consent for the use of blood in the research was obtained from all donors. All experiments were conducted in accordance with the Fundamental Guidelines for Proper Conduct of Animal Experiment and Related Activities in Academic Research Institutions under the jurisdiction of the Ministry of Education, Culture, Sports, Science and Technology, Japan.

### Parasites

The *Plasmodium falciparum* 3D7, Dd2, HB3 clones were obtained from the Malaria Research and Reference Reagent Resource Center (MR4; American Type Culture Collection, Manassas, VA) and were cultured in 25-cm tissue flasks with RPMI-1640 medium (Sigma-Aldrich, Tokyo, Japan) supplemented with 25 mM HEPES, 0.15% sodium carbonate, 0.5% Albumax II (Invitrogen, Carlsbad, CA, USA), 100 μM hypoxanthine, and 12.5 μg/ml gentamycin (Sigma-Aldrich). The parasites were maintained in human erythrocytes (hematocrit 1%) with daily replacement of 10 ml of fresh medium and inoculated in a 37 °C atmosphere containing a 5% CO_2_/3% O_2_/balanced N_2_ gas mixture. At peak parasitemia, the packed erythrocytes were harvested, washed once with RPMI medium, and then subjected to MACS magnetic separation (Miltenyl Biotec, Auburn, CA, USA) to enrich the purity of iRBCs of trophozoite and schizont stages to >85% as early described^28^. Thereafter, the iRBCs were washed once with RPMI-1640 medium and used for the phagocytic assay.

### Macrophages and the phagocytic assay

Human mononuclear cells were separated from the peripheral blood of healthy donors by using Ficoll-Paque™ PLUS (GE Healthcare) and were cultured for 8 days, at 37 °C in a humidified atmosphere containing 5% CO_2,_ in RPMI-1640 medium (Sigma-Aldrich) supplemented with human recombinant granulocyte-macrophage colony-stimulating factor (GM-CSF) and human recombinant interferon gamma (IFN-γ) (R&D system) for cell differentiation. Addition of these cytokines to mononuclear cells cultures aids in differentiation of effector macrophages^29^. All cells were prepared from the same donor to minimize nonspecific reactions of macrophages to the erythrocytes. Briefly, blood diluted with warm PBS (1:1) was layered onto Ficoll and centrifuged at 800 *g* for 30 minutes at 21 °C. The peripheral blood mononuclear cell (PBMC) layer was harvested, washed twice with PBS, and then resuspended to 1 × 10^6^ cells/ml in RPMI-1640 supplemented with 10% heat-inactivated fetal calf serum and 25 mg/L penicillin/streptomycin. The cells were pipetted onto 25-cm flasks and initially incubated for 3 hours. Thereafter, non-adherent cells were removed and the adherent cells were cultured in medium containing 20 ng/ml GM-CSF and 100 ng/ml IFN-γ for 7 days at 37 °C (the medium was replaced with fresh medium after 3 days of culture) to allow differentiation into macrophages. Macrophages were detached by treatment with 1% trypsin-EDTA solution for 10 min, washed with ice-cold PBS and RPMI medium, counted, and then seeded onto 6-well culture plates at 5 × 10^6^ or cover slides at 1 × 10^5^ for the phagocytic assay. Macrophages were cultured with synchronized-iRBCs, saponin-treated iRBCs, or human non-infected RBCs at a ratio of 1:20 at 37 °C in *Plasmodium-*culturing medium^10^. To determine the percentage of phagocytosis, cultured macrophages on the cover slides were washed twice with PBS, fixed in absolute methanol for 10 min, stained with 10% Giemsa solution for 30 min, and then counted by microscopic examination using an oil immersion lens. The apparent malaria pigment (homozoin) and iRBCs within the macrophages were used as determinants of phagocytosis. In addition, the ability of differentiated macrophages, monocytes, and the human monocyte cell line THP1 (RIKEN, Saitama, Japan) to eradicate *P. falciparum* parasites was determined *in vitro*. Briefly, 1 × 10^5^ phagocytic cells were cultured with enriched-iRBCs (ratio, 1:20) in a 96-well plate for 2 h. Then, the cells were lysed by 2 cycles of freezing and thawing and their supernatants were then examined by using an ELISA kit (Malaria Ag CELISA; Cellabs, Sydney, New South Wales, Australia) according to the manufacturer’s instructions.

### RNA isolation and integrity

Adherent macrophages cultured for 2 h with enriched-iRBCs, saponin-treated iRBCs, or human non-infected RBC (n = 3) were washed with ice-cold PBS and lysed with TRIzol Reagent (Invitrogen) for RNA extraction. Chloroform-isoamyl alcohol (5:1) was added to the lysed cells, which were then vortexed and centrifuged for 15 min at 12,000 × *g* at 4 °C. The aqueous layer was subjected to RNA extraction by using RNeasy Mini kit columns (Qiagen, Hilden, Germany) according to the manufacturer’s instructions. RNA integrity was assessed by determining UV 260/280 absorbance ratios and by examining 28S/18S ribosomal RNA bands with an Agilent 2100 bioanalyzer (Agilent Technologies, Santa Clara, CA, USA) according to the manufacturer’s instructions. Only high-quality RNA samples (integrity value > 7.0) were used for the microarray analysis.

### Microarray analysis and bioinformatics

Microarray analysis was conducted as previously described^14^. Briefly, cy3-labeled complementary RNA probe synthesis was initiated with 50 ng of total RNA by using the Agilent Low Input Quick Amp Labeling kit, one color (Agilent Technologies). Agilent SurePrint G3 Human GE 8 × 60 K microarrays (G4851) were used according to the manufacturer’s instructions. Slides were scanned with an Agilent’s High-Resolution Microarray Scanner, and image data were processed by using Agilent Feature Extraction software, version 10.7.3.1. All data were subsequently uploaded into GeneSpring GX, version 12.6 for data analysis. Each gene expression array dataset was normalized to the *in silico* pool for the macrophages cultured with RBCs. Statistically significant differences in gene expression between macrophages cultured with RBCs and those cultured with iRBCs were determined by using a moderated T-test (*P* < 0.05). Genes that showed at least a 2.0-fold change in their expression with a statistical significant difference (*P* < 0.05) were assigned to a Gene Ontology group in the DAVID Bioinformatics Database (http://david.abcc.ncifcrf.gov/home.jsp). GO analysis was used to classify the genes based on their known functions^14^. For all analyses, *P* values were calculated by using the Fisher’s exact test and considered to be significant at *P* < 0.05. The microarray data are publically available at the Gene Expression Omnibus (GEO) database (http: www.ncbi.nlm.nih.gov/geo/) under the accession number GSE77122.

### Quantitative real-time polymerase chain reaction (qRT–PCR)

Total RNA was reverse transcribed to first-strand cDNA (Invitrogen) using oligo-dT primer, according to manufacturer’s instructions. The expression of targeted genes was analyzed with qRT–PCR using an ABI Prism Genetic Analyzer (Applied Biosystems, Carlsbad, CA, USA) with SYBR Green (Applied Biosystems) and the specific primers listed in the Supplemental Table (Table S3). The primers for qRT–PCR were designed with the Primer Express software (Applied Biosystems). Specific gene expression was normalized to the expression of ubiquitin, by using the β-actin housekeeping gene. Relative gene expression was calculated with the 2^−ΔΔCt^ method and the fold-increase was determined based on the gene expression of macrophages cultured with RBCs.

### Immunofluorescence assay

Adherent differentiated macrophages on round glass coverslips in 24-well plastic plates were incubated with iRBCs for phagocytosis. After a 2-h incubation, the coverslips were washed three times with PBS and fixed with 4% paraformaldehyde solution for 20 min. Cells were permeabilized with 0.3 triton X100 (Sigma) in PBS for 5 min, and then incubated with the primary antibody (anti-DEFB130; Santa Cruz Biotechnology, CA, USA) diluted in 3% fetal calf serum in PBS (1:100) for 1 h at 37 °C in a moist chamber. A secondary antibody of Alexa-Fluor® 488-conjugated goat anti-rabbit IgG (Molecular Probes, Invitrogen, Carlsbad, CA, USA) at a dilution of 1:400 was applied to the coverslips and incubated for 30 min at 37 °C. Hoechst was used to label the nuclear DNA of the cells (Molecular Probes). Coverslips were mounted (Dako, Denmark) and analyzed by use of a confocal laser scanning microscope (TCS NT, Leica, Heidelberg, Germany).

### ELISA

Detection of DEFB130 and malarial antigen (parasite biomass) was performed by using commercial ELISA kits (DEFB130; Cusabio Biotech, Wuhan, China and Malaria Ag CELISA; Cellabs, Sydney, New South Wales, Australia).

### esiRNA-mediated knockdown

Differentiated macrophages were transfected with esiRNA against DEFB130 or negative esiRNA control (both from Sigma-Aldrich) with INTERFERin Transfection Reagent (Polyplus, Illkirch, France) according to the manufacturer’s instructions. Transfection reactions were performed in RPMI1640-serum-free medium (Sigma). Half of medium was changed after 4 h and the whole medium was changed 20 h after transfection. Cells were harvested 48 h after transfection for protein analysis by ELISA (Cusabio Biotech) and growth inhibition assay (only for cells with 55% or above transfection efficacy). To perform growth inhibitory assay, transfected macrophages were co-cultured with enriched iRBCs for 2 h. Thereafter, cells were lysed by 2 cycles of freezing and thawing and their supernatants were examined by using ELISA kits for parasite biomass (CELISA, Cellabs) and for detecting DEFB130 (Cusabio Biotech). Before lysing the cells, 10 μl of iRBCs were transferred into new 96-well plate, and mixed with 90 μl fresh RBCs and medium at hematocrit of 2%. Cultures were incubated for 24 h and the parasite biomass was determined by ELISA (CELISA, Cellabs).

### Transfection of the THP1 cell line

The coding region of DEFB130 was amplified from cDNA and subcloned into the P3XFLAG-CMV14 mammalian expression vector (Sigma) using the following specific primer set: 5′-TGAATTCATGAAACTCCATTCCCTTAT-3′, which includes an *Eco*RI restriction enzyme site (underlined), and 5′-AGGATCCTTGAGGAGATTTTCCTTTGG-3′, which includes a *Bam*HI restriction enzyme site (underlined). Transfection was performed using the Amaxa cell line nucleofector kit V and the Amaxa nucleofector II device (Lonza, ME, USA), according to the manufacturer’s instructions. Briefly, THP1 monocyte cell line cells, cultured in RPMI-1640 supplemented with 10% heat-inactivated fetal calf serum and 25 mg/L penicillin/streptomycin, were harvested, washed once with RPMI-1640 medium, and counted for transfection. The 2 × 10^6^ cells were resuspended in 100 μl of nucleofector solution containing 2 μg of plasmids and then transferred to a nucleoporation cuvette. Electroporation was performed in a nucleofector II device using the V001 program. The cells were cultured for 24 h and then analyzed for overexpression phenotypes by use of qRT-PCR and ELISA. Finally, the ability of the cells to inhibit the growth of parasites/reduce the parasite biomass was assessed after culturing them with enriched-iRBCs for 2 h and using a malaria ELISA kit.

### Peptides

DEFB130 peptide (GVIPGQKQCIALKGVCRDKLCSTLDDTIGICNEGKKCCRRWWILEPYPTPVPKGKSP), scrambled DEFB130 (sDEFB130) (PSKGKPVPTPYPELIWWRRCCKKGENCIGITDDLTSCLKDRCVGKLAICQKQGPIVG), N-terminal DEFB130 (QCIALKGVCRDKLCSTLDDTIGICNEGKKCCR), and C-terminal DEFB130 (ILEPYPTPVPKGKSP) were synthesized and obtained from Eurofins Scientific (Tokyo, Japan) at a purity grade of >95%. The peptides were dissolved in PBS to obtain stock solutions of 1 mg ml^−1^.

### Measurement of the CD spectra of recombinant DEFB130 and 3D structure modeling

Recombinant DEFB130 was produced in *Escherichia coli* as a 6xHistidine-tag fusion protein. Briefly, the coding region was amplified from cDNA prepared from human macrophages with the following specific primer set: 5′-AGGATCCGGCGTTATTCCAGGACAAAA-3′, which includes a *Bam*HI restriction enzyme site (underlined), and 5′-ACTCGAGTTAAGGAGATTTTCCTTTG-3′, which includes a *Xho*I restriction enzyme site (underlined). PCR products were digested with the appropriate restriction enzymes and then ligated into a pET-28a expression vector (Novagen), which had been digested with the same restriction enzymes. Recombinant protein was expressed in large-scale *E. coli* DH5α strain cultures (Takara Bio Inc., Osaka, Japan) and purified by means of Ni-NTA affinity chromatography, according to the manufacturer’s instructions (Qiagen, Hilden, Germany). The purified recombinant protein was dialyzed in PBS, subjected to sodium dodecyl sulphate polyacrylamide gel electrophoresis (SDS-PAGE), and stained with Coomassie Brilliant Blue. The recombinant DEFB130 was stored at 4 °C until use. To measure the Circular Dichroism (CD) spectra, 150 μg/ml of recombinant DEFB130 and synthetic peptide were prepared in appropriate buffer (pH 7.4), and the CD spectra were measured with a Jasco J-820 Circular dichroism dispersion meter in a 0.1-cm quartz cell at room temperature^19^. The spectra were the averages of eight consecutive scans from 260 to 195 nm, recorded with a bandwidth of 1 nm, a time constant of 4 sec, and a scan rate of 20 nm/min. The 3D structure modeling of DEFB130 was performed by using SWISS-MODEL^30^ and modeling structures of DEFB3 (1KJ6)^31^. The electrostatic surface potentials were calculated by using the program APBS^32^ and are represented by PyMOL (www.pymol.org).

### Parasite proliferation assay

Unsynchronized *P. falciparum* 3D7, Dd2, and HB3 clones (MR4) were cultured for 72 h in the absence or presence of peptides in 96-well plates with a starting parasitemia of 1% and a hematocrit of 2%. The IC_50_ values for the synthetic peptides were determined by using SYBR Green I (Lonza) as described previously^33^. To assay the hemolytic activity of the peptides, fresh RBCs at 2.5% hematocrit were washed in PBS, resuspended in 100 μl of PBS containing increasing concentrations of each peptide in 96-well microtiter plates, and inoculated at 37 °C for 2 h^27^. Then, the RBCs were pelleted and the supernatant was harvested and diluted 1:10 in PBS to measure the release of hemoglobin from the RBCs at 405 nm. The hemolytic activity calculation was based on the value for control RBCs lysed with distilled water; all values were determined in triplicate.

### Mice and infection

Specific-pathogen-free 7-week-old female C57BL/6 mice purchased from Clea (Tokyo, Japan) were infected with *Plasmodium yoelii* 17-XNL via intraperitoneal inoculation of 1 × 10^7^ fresh iRBCs from donor mice. Five mice per group were treated by intravenous injection (5 mg/kg dose) with DEFB130, sDEFB130, or PBS on days 4, 5, and 6 post-infection. Parasitemias were determined by microscopy of methanol-fixed thin blood smears stained with 10% Giemsa solution.

### Statistical analysis

Statistical analysis was performed using GraphPad Prism 5 software (GraphPad Software Inc., La Jolla, CA, USA); results were considered statistically significant when *P* < 0.05. Student’s t test was used to compare the differences between two independent groups and one-way ANOVA followed by Tukey’s multiple comparisons procedure was used to compare the differences among groups.

## Additional Information

**How to cite this article**: Terkawi, M. A. *et al*. Involvement of ß-defensin 130 (DEFB130) in the macrophage microbicidal mechanisms for killing *Plasmodium falciparum. Sci. Rep.* **7**, 41772; doi: 10.1038/srep41772 (2017).

**Publisher's note:** Springer Nature remains neutral with regard to jurisdictional claims in published maps and institutional affiliations.

## Supplementary Material

Supplementary Information

## Figures and Tables

**Table 1:** Selected up-regulated genes of macrophages phagocytizing iRBC as determined by DNA microarray analysis and qRT-PCR.

Gene	Genbank Accession number	Fold changea^a^		
		Microarray	qRT-PCR^b^	qRT-PCR^c^
*SMTNL2*	NM_198501	4.613	7.20 ± 8.59	14.57 ± 11.72
*DEFB130*	NM_001037804	4.569	4.49 ± 3.93	3.45 ± 1.75
*RIT2*	NM_002930	4.543	5.74 ± 2.53	4.11 ± 6.60
*SYTL4*	NM_080737	4.459	5.87 ± 3.77	6.20 ± 4.60
*PRSS41*	NM_001135086	3.912	1.71 ± 0.85	3.44 ± 3.06
*GRP*	NM_002091	2.726	4.62 ± 4.95	3.38 ± 1.80
*ADAM19*	NM_033274	2.209	1.54 ± 1.30	1.72 ± 0.73

^a^Fold change indicates the mean expression level of the gene in macrophages co-cultured with iRBCs normalized to that in macrophages co-cultured with RBCs.

^b^Results of qRT-PCR are from the same samples used for the microarray. Results represent the mean fold change ± SEM of three independent experiments.

^c^Results of qRT-PCR are from pooled data of samples collected from three individual donors. Results represent the mean fold change ± SEM (n = 3).

**Table 2:** Antimalarial activity of DEFB130.

	Parasite strains (IC_50_ μM)		
	3D7	Dd2	HB3
DEFB130	47.12 ± 2.22	43.53 ± 3.81	49.22 ± 3.16
sDEFB130	>200	>200	>200
Nt-DEFB130	93.02 ± 0.88	91.31 ± 2.09	90.55 ± 1.63
Ct-DEFB130	>200	>200	>200

The IC_50_ values of synthetic DEFB130, scrambled peptide, and N-terminal and C-terminal domain peptides against different strains of *P. falciparum* parasites *in vitro*.
