# Supplementary material for: Involvement of β-defensin 130 (DEFB130) in the macrophage microbicidal mechanisms for killing Plasmodium falciparum
Source: Sci Rep. 2017 Feb 9;7:41772. doi: 10.1038/srep41772 (PMC5299406; doi:10.1038/srep41772)
Supplement: Supplementary Information [file srep41772-s1.pdf]

Supplementary Information

Involvement of β-defensin 130 (DEFB130) in the macrophage microbicidal mechanisms for killing *Plasmodium falciparum*

Mohamad Alaa Terkawi[1,2], Ryo Takano[1], Atsushi Furukawa[3], Fumi Murakoshi[1], and Kentaro Kato[1,4]*

[1]National Research Center for Protozoan Diseases, Obihiro University of Agriculture and Veterinary Medicine, Inada-cho, Obihiro, Hokkaido 080-8555, Japan.

[2]Frontier Research Center for Advanced Material and Life Science, Department of Orthopedic Surgery, School of Medicine, Hokkaido University, Kita 21, Nishi 11, Kita-ku, Sapporo, Hokkaido 001-0021, Japan.

[3]Laboratory of Biomolecular Science, Faculty of Pharmaceutical Sciences, Hokkaido University, Kita-12, Nishi-6, Kita-ku, Sapporo 060-0812, Japan.

[4]Research Center for Global Agromedicine, Obihiro University of Agriculture and Veterinary Medicine, Inada-cho, Obihiro, Hokkaido 080-8555, Japan.

* Correspondence and requests for materials should be addressed to Dr. Kentaro Kato,
National Research Center for Protozoan Diseases, Obihiro University of Agriculture and Veterinary Medicine, Inada-cho, Obihiro, Hokkaido 080-8555, Japan

Tel: +81-155-49-5645
Fax: +81-155-49-5646
E-mail: kkato@obihiro.ac.jp

Supplementary Table S1. Genes significantly differentially down-regulated in the macrophages phagocytizing iRBCs compared with those in macrophages phagocytizing RBCs.

#	Probe Name	*P* value	Fold change [a]	Gene Symbol	Description	Genbank Accession
1	A_23_P203558	0.0000	75.43295	HBB	Homo sapiens hemoglobin, beta (HBB), mRNA [NM_000518]	NM_000518
2	A_33_P3258362	0.0000	57.840027	HBA2	Homo sapiens hemoglobin, alpha 2 (HBA2), mRNA [NM_000517]	NM_000517
3	A_24_P75190	0.0000	41.79774	HBD	Homo sapiens hemoglobin, delta (HBD), mRNA [NM_000519]	NM_000519
4	A_23_P26457	0.0000	40.554462	HBA2	Homo sapiens hemoglobin, alpha 2 (HBA2), mRNA [NM_000517]	NM_000517
5	A_19_P00315798	0.0251	13.765166	LOC100506374	PREDICTED: Homo sapiens hypothetical LOC100506374 (LOC100506374), miscRNA [XR_109498]	XR_109498
6	A_23_P403898	0.0425	10.078446	PTPN3	Homo sapiens protein tyrosine phosphatase, non-receptor type 3 (PTPN3), transcript variant 1, mRNA [NM_002829]	NM_002829
7	A_33_P3401156	0.0215	9.821612	ETV1	Homo sapiens ets variant 1 (ETV1), transcript variant 1, mRNA [NM_004956]	NM_004956
8	A_23_P103877	0.0003	8.578429	LRRC38	Homo sapiens leucine rich repeat containing 38 (LRRC38), mRNA [NM_001010847]	NM_001010847
9	A_33_P3254844	0.0419	8.3073635	CEACAM7	Homo sapiens carcinoembryonic antigen-related cell adhesion molecule 7 (CEACAM7), mRNA [NM_006890]	NM_006890
10	A_24_P392151	0.0267	8.011117	C11orf86	Homo sapiens chromosome 11 open reading frame 86 (C11orf86), mRNA [NM_001136485]	NM_001136485
11	A_23_P31143	0.0037	6.489389	TPD52L1	Homo sapiens tumor protein D52-like 1 (TPD52L1), transcript variant 2, mRNA [NM_001003395]	NM_001003395
12	A_23_P112554	0.0319	6.0272794	COL15A1	Homo sapiens collagen, type XV, alpha 1 (COL15A1), mRNA [NM_001855]	NM_001855
13	A_24_P290751	0.0463	5.8838143	DTX1	Homo sapiens deltex homolog 1 (Drosophila) (DTX1), mRNA [NM_004416]	NM_004416
14	A_24_P286951	0.0331	5.74825	C10orf81	Homo sapiens chromosome 10 open reading frame 81 (C10orf81), transcript variant 4, mRNA [NM_024889]	NM_024889
15	A_23_P86532	0.0023	5.4387827	BICC1	bicaudal C homolog 1 (Drosophila) [Source:HGNC Symbol;Acc:19351] [ENST00000263103]	AK026129
16	A_24_P58488	0.0382	5.4361105	C2orf73	Homo sapiens chromosome 2 open reading frame 73 (C2orf73), mRNA [NM_001100396]	NM_001100396
17	A_24_P142305	0.0000	5.12649	HBA2	Homo sapiens hemoglobin, alpha 2 (HBA2), mRNA [NM_000517]	NM_000517
18	A_33_P3408514	0.0414	5.088986	SCNN1D	Homo sapiens sodium channel, nonvoltage-gated 1, delta (SCNN1D), transcript variant 1, mRNA [NM_001130413]	NM_001130413
19	A_33_P3692979	0.0036	4.8503685	LOC283485	Homo sapiens cDNA FLJ36543 fis, clone TRACH2006194. [AK093862]	AK093862
20	A_33_P3274754	0.0355	4.623998	LOC100131434	Homo sapiens uncharacterized LOC100131434 (LOC100131434), non-coding RNA [NR_027455]	NR_027455
21	A_23_P123488	0.0327	4.4949994	PRDM14	Homo sapiens PR domain containing 14 (PRDM14), mRNA [NM_024504]	NM_024504
22	A_33_P3242548	0.0276	4.462692	PRDM5	Homo sapiens PR domain containing 5 (PRDM5), mRNA [NM_018699]	NM_018699
23	A_23_P103803	0.0415	4.342108	FCRL3	Homo sapiens Fc receptor-like 3 (FCRL3), mRNA [NM_052939]	NM_052939
24	A_24_P293530	0.0067	4.082462	CYP4X1	Homo sapiens cytochrome P450, family 4, subfamily X, polypeptide 1 (CYP4X1), mRNA [NM_178033]	NM_178033
25	A_32_P124708	0.0394	3.9461558	ONECUT2	Homo sapiens one cut homeobox 2 (ONECUT2), mRNA [NM_004852]	NM_004852
26	A_33_P3408047	0.0071	3.8830614		PREDICTED: Homo sapiens hypothetical protein LOC100289308 (LOC100289308), mRNA [XM_002344174]	XM_002344174

27	A_23_P57474	0.0277	3.6703427	OSBP2	Homo sapiens oxysterol binding protein 2 (OSBP2), transcript variant 1, mRNA [NM_030758]	NM_030758
28	A_23_P250607	0.0009	3.6646404	PLS3	Homo sapiens plastin 3 (PLS3), transcript variant 1, mRNA [NM_005032]	NM_005032
29	A_33_P3406866	0.0456	3.6627853	NFASC	Homo sapiens neurofascin (NFASC), transcript variant 1, mRNA [NM_001005388]	NM_001005388
30	A_32_P1712	0.0025	3.6582713	RNASE2	Homo sapiens ribonuclease, RNase A family, 2 (liver, eosinophil-derived neurotoxin) (RNASE2), mRNA [NM_002934]	NM_002934
31	A_23_P113553	0.0372	3.6476438	MAGEA11	Homo sapiens melanoma antigen family A, 11 (MAGEA11), transcript variant 2, mRNA [NM_001011544]	NM_001011544
32	A_33_P3302275	0.0316	3.6298826	CXorf66	Homo sapiens chromosome X open reading frame 66 (CXorf66), mRNA [NM_001013403]	NM_001013403
33	A_23_P92909	0.0056	3.6233866	SPINK6	Homo sapiens serine peptidase inhibitor, Kazal type 6 (SPINK6), transcript variant 1, mRNA [NM_205841]	NM_205841
34	A_23_P349966	0.0036	3.4515724	TMEM130	Homo sapiens transmembrane protein 130 (TMEM130), transcript variant 2, mRNA [NM_152913]	NM_152913
35	A_33_P3412798	0.0369	3.4070928	FRG2B	Homo sapiens FSHD region gene 2 family, member B (FRG2B), mRNA [NM_001080998]	NM_001080998
36	A_23_P17190	0.0037	3.3890638	KBTBD10	Homo sapiens kelch repeat and BTB (POZ) domain containing 10 (KBTBD10), mRNA [NM_006063]	NM_006063
37	A_33_P3423791	0.0272	3.368297	GRHL1	Homo sapiens grainyhead-like 1 (Drosophila) (GRHL1), mRNA [NM_198182]	NM_198182
38	A_24_P626932	0.0396	3.3487058	MUC3	Homo sapiens SIB 276 intestinal mucin (MUC3) mRNA, partial cds. [AF007191]	AF007191
39	A_33_P3240249	0.0332	3.332887	LOC100129125	Homo sapiens cDNA FLJ43724 fis, clone TESOP2007688. [AK125712]	AK125712
40	A_33_P3725227	0.0260	3.2845643	COBL	Homo sapiens cordon-bleu homolog (mouse) (COBL), mRNA [NM_015198]	NM_015198
41	A_23_P1331	0.0476	3.2607222	COL13A1	Homo sapiens collagen, type XIII, alpha 1 (COL13A1), transcript variant 5, mRNA [NM_080801]	NM_080801
42	A_24_P822931	0.0494	3.229193	LOC100329135	Homo sapiens uncharacterized LOC100329135 (LOC100329135), transcript variant 1, mRNA [NM_001195576]	NM_001195576
43	A_33_P3258003	0.0175	3.1709845	ANKS1B	Homo sapiens ankyrin repeat and sterile alpha motif domain containing 1B (ANKS1B), transcript variant 1, mRNA [NM_152788]	NM_152788
44	A_33_P3215282	0.0002	3.1573417	TTBK1	Homo sapiens tau tubulin kinase 1 (TTBK1), mRNA [NM_032538]	NM_032538
45	A_33_P3379535	0.0014	3.0833657		Homo sapiens cDNA FLJ45698 fis, clone FEBRA2017811. [AK127601]	AK127601
46	A_24_P165864	0.0329	3.0750778	P2RY14	Homo sapiens purinergic receptor P2Y, G-protein coupled, 14 (P2RY14), transcript variant 2, mRNA [NM_014879]	NM_014879
47	A_32_P711043	0.0096	3.073225		Uncharacterized proteincDNA FLJ40144 fis, clone TESTI2013012 [Source:UniProtKB/TrEMBL;Acc:Q8N811] [ENST00000326348]	AK097463
48	A_32_P154342	0.0075	3.062463	SLCO4C1	Homo sapiens solute carrier organic anion transporter family, member 4C1 (SLCO4C1), mRNA [NM_180991]	NM_180991
49	A_23_P331560	0.0192	3.056756	SLC26A4	Homo sapiens solute carrier family 26, member 4 (SLC26A4), mRNA [NM_000441]	NM_000441
50	A_33_P3359973	0.0212	3.0529284		BX109507 Soares_NFL_T_GBC_S1 Homo sapiens cDNA clone IMAGp998I133863, mRNA sequence [BX109507]	BX109507
51	A_23_P216225	0.0451	3.0459886	EGR3	Homo sapiens early growth response 3 (EGR3), transcript variant 1, mRNA [NM_004430]	NM_004430
52	A_23_P254741	0.0260	3.0299306	SOD3	Homo sapiens superoxide dismutase 3, extracellular (SOD3), mRNA [NM_003102]	NM_003102
53	A_33_P3390918	0.0082	3.018749	FSD2	Homo sapiens fibronectin type III and SPRY domain containing 2 (FSD2), mRNA	NM_001007122

					[NM_001007122]	
54	A_33_P3224078	0.0387	3.013545	LOC442421	Homo sapiens uncharacterized LOC442421 (LOC442421), non-coding RNA [NR_024496]	NR_024496
55	A_23_P396934	0.0498	3.0120006	KIAA1239	Homo sapiens KIAA1239 (KIAA1239), mRNA [NM_001144990]	NM_001144990
56	A_19_P00319981	0.0480	2.952404	LOC100506119	PREDICTED: Homo sapiens hypothetical LOC100506119, transcript variant 1 (LOC100506119), miscRNA [XR_108951]	XR_108951
57	A_23_P32500	0.0207	2.919216	STAB1	Homo sapiens stabilin 1 (STAB1), mRNA [NM_015136]	NM_015136
58	A_33_P3362311	0.0424	2.8549187	C6orf222	Homo sapiens chromosome 6 open reading frame 222 (C6orf222), mRNA [NM_001010903]	NM_001010903
59	A_33_P3322999	0.0098	2.8259497	C10orf105	Homo sapiens chromosome 10 open reading frame 105 (C10orf105), transcript variant 1, mRNA [NM_001164375]	NM_001164375
60	A_33_P3240727	0.0013	2.7739053	ZNF697	Homo sapiens zinc finger protein 697 (ZNF697), mRNA [NM_001080470]	NM_001080470
61	A_24_P357847	0.0016	2.769883		immunoglobulin kappa variable 3-15 [Source:HGNC Symbol;Acc:5816] [ENST00000390252]	XM_003403505
62	A_24_P12397	0.0457	2.758812	TREM2	Homo sapiens triggering receptor expressed on myeloid cells 2 (TREM2), mRNA [NM_018965]	NM_018965
63	A_33_P3397920	0.0030	2.7501643		PREDICTED: Homo sapiens hypothetical LOC730184 (LOC730184), miscRNA [XR_109942]	XR_109942
64	A_33_P3230166	0.0362	2.7258637	NALCN	Homo sapiens sodium leak channel, non-selective (NALCN), mRNA [NM_052867]	NM_052867
65	A_33_P3216610	0.0116	2.721196	TMPRSS4	Homo sapiens transmembrane protease, serine 4 (TMPRSS4), transcript variant 1, mRNA [NM_019894]	NM_019894
66	A_23_P75283	0.0162	2.716025	RBP4	Homo sapiens retinol binding protein 4, plasma (RBP4), mRNA [NM_006744]	NM_006744
67	A_23_P106874	0.0041	2.7159984	PMFBP1	Homo sapiens polyamine modulated factor 1 binding protein 1 (PMFBP1), transcript variant 1, mRNA [NM_031293]	NM_031293
68	A_33_P3301920	0.0278	2.7113001	USP50	Homo sapiens ubiquitin specific peptidase 50 (USP50), mRNA [NM_203494]	NM_203494
69	A_24_P272313	0.0234	2.7098494	C2orf55	Homo sapiens chromosome 2 open reading frame 55 (C2orf55), mRNA [NM_207362]	NM_207362
70	A_33_P3294985	0.0153	2.6578007	JPX	Homo sapiens JPX transcript, XIST activator (non-protein coding) (JPX), non-coding RNA [NR_024582]	NR_024582
71	A_23_P3379	0.0095	2.6561167	RASGRF1	Homo sapiens Ras protein-specific guanine nucleotide-releasing factor 1 (RASGRF1), transcript variant 1, mRNA [NM_002891]	NM_002891
72	A_33_P3239654	0.0104	2.6376486	HCG4B	Homo sapiens cDNA FLJ32165 fis, clone PLACE6000424. [AK056727]	AK056727
73	A_23_P378926	0.0474	2.6287177	ADRA2B	Homo sapiens adrenergic, alpha-2B-, receptor (ADRA2B), mRNA [NM_000682]	NM_000682
74	A_33_P3310744	0.0051	2.6228724	TTC34	Homo sapiens tetratricopeptide repeat domain 34 (TTC34), mRNA [NM_001242672]	NM_001242672
75	A_23_P304716	0.0076	2.6103628	HES2	Homo sapiens hairy and enhancer of split 2 (Drosophila) (HES2), mRNA [NM_019089]	NM_019089
76	A_33_P3605529	0.0257	2.6088846	LOC400548	Homo sapiens uncharacterized LOC400548 (LOC400548), non-coding RNA [NR_033984]	NR_033984
77	A_23_P56578	0.0241	2.6034815	VIT	Homo sapiens vitrin (VIT), transcript variant 1, mRNA [NM_053276]	NM_053276
78	A_33_P3232478	0.0017	2.601206	CACNA2D1	Homo sapiens calcium channel, voltage-dependent, alpha 2/delta subunit 1 (CACNA2D1), mRNA [NM_000722]	NM_000722
79	A_33_P3289296	0.0474	2.576404	TMEM37	Homo sapiens transmembrane protein 37 (TMEM37), mRNA [NM_183240]	NM_183240
80	A_23_P133123	0.0149	2.573241	MND1	Homo sapiens meiotic nuclear divisions 1 homolog (S. cerevisiae) (MND1), mRNA [NM_032117]	NM_032117
81	A_19_P00317885	0.0178	2.5692213	XLOC_002613	DA963975 STOMA2 Homo sapiens cDNA clone STOMA2001973 5', mRNA sequence	DA963975

					[DA963975]	
82	A_23_P209055	0.0091	2.5651057	CD22	Homo sapiens CD22 molecule (CD22), transcript variant 1, mRNA [NM_001771]	NM_001771
83	A_24_P32574	0.0444	2.5645182	KNDC1	Homo sapiens kinase non-catalytic C-lobe domain (KIND) containing 1 (KNDC1), transcript variant 1, mRNA [NM_152643]	NM_152643
84	A_24_P62783	0.0317	2.5482633	FABP3	Homo sapiens fatty acid binding protein 3, muscle and heart (mammary-derived growth inhibitor) (FABP3), mRNA [NM_004102]	NM_004102
85	A_33_P3411907	0.0005	2.5408232	FGF5	Homo sapiens fibroblast growth factor 5 (FGF5), transcript variant 2, mRNA [NM_033143]	NM_033143
86	A_32_P118372	0.0356	2.539435	INTU	Homo sapiens inturned planar cell polarity effector homolog (Drosophila) (INTU), mRNA [NM_015693]	NM_015693
87	A_23_P85269	0.0134	2.5359707	TTN	Homo sapiens titin (TTN), transcript variant N2-A, mRNA [NM_133378]	NM_133378
88	A_33_P3227086	0.0055	2.535094		Homo sapiens cDNA FLJ38343 fis, clone FCBBF3028472. [AK095662]	AK095662
89	A_33_P3382803	0.0094	2.527198	FLJ43390	Homo sapiens uncharacterized LOC646113 (FLJ43390), non-coding RNA [NR_015358]	NR_015358
90	A_33_P3379922	0.0075	2.5157833	PROC	Homo sapiens protein C (inactivator of coagulation factors Va and VIIIa) (PROC), mRNA [NM_000312]	NM_000312
91	A_33_P3379039	0.0371	2.5151517	IGLL5	Homo sapiens immunoglobulin lambda-like polypeptide 5 (IGLL5), transcript variant 1, mRNA [NM_001178126]	NM_001178126
92	A_23_P400378	0.0142	2.4985473	GPBAR1	Homo sapiens G protein-coupled bile acid receptor 1 (GPBAR1), transcript variant 1, mRNA [NM_001077191]	NM_001077191
93	A_23_P161135	0.0040	2.492778	LEPR	Homo sapiens leptin receptor (LEPR), transcript variant 1, mRNA [NM_002303]	NM_002303
94	A_23_P151133	0.0203	2.4827256	TSPAN9	Homo sapiens tetraspanin 9 (TSPAN9), transcript variant 1, mRNA [NM_006675]	NM_006675
95	A_23_P251412	0.0385	2.4623108	SCGN	Homo sapiens secretagogin, EF-hand calcium binding protein (SCGN), mRNA [NM_006998]	NM_006998
96	A_23_P381351	0.0362	2.461603	CSH1	Homo sapiens chorionic somatomammotropin hormone 1 (placental lactogen) (CSH1), mRNA [NM_001317]	NM_001317
97	A_33_P3260747	0.0157	2.4597104	NHSL2	NHS-like 2 [Source:HGNC Symbol;Acc:33737] [ENST00000373677]	BC033261
98	A_33_P3323029	0.0431	2.4457939	LOC400965	Homo sapiens cDNA FLJ40699 fis, clone THYMU2025572. [AK098018]	AK098018
99	A_33_P3415551	0.0338	2.4406023	GPAT2	Homo sapiens glycerol-3-phosphate acyltransferase 2, mitochondrial (GPAT2), nuclear gene encoding mitochondrial protein, mRNA [NM_207328]	NM_207328
100	A_33_P3418838	0.0164	2.4362655	TPD52	tumor protein D52 [Source:HGNC Symbol;Acc:12005] [ENST00000523564]	XM_001716081
101	A_33_P3367392	0.0046	2.4058216	FAM167B	Homo sapiens family with sequence similarity 167, member B (FAM167B), mRNA [NM_032648]	NM_032648
102	A_23_P344421	0.0143	2.4019687	ROBO4	Homo sapiens roundabout homolog 4, magic roundabout (Drosophila) (ROBO4), mRNA [NM_019055]	NM_019055
103	A_24_P778906	0.0264	2.3986723			XR_110942
104	A_24_P626951	0.0320	2.394688		immunoglobulin kappa variable 1-16 [Source:HGNC Symbol;Acc:5732] [ENST00000479981]	AF078945
105	A_24_P64653	0.0132	2.3940938	METTL7B	Homo sapiens methyltransferase like 7B (METTL7B), mRNA [NM_152637]	NM_152637
106	A_23_P71855	0.0006	2.3838327	C5	Homo sapiens complement component 5 (C5), mRNA [NM_001735]	NM_001735
107	A_33_P3318606	0.0396	2.3820047	SHC2	Homo sapiens SHC (Src homology 2 domain containing) transforming protein 2, mRNA (cDNA clone IMAGE:4752153), with apparent retained intron. [BC034544]	BC034544

108	A_33_P3319845	0.0044	2.356721	OR51B6	Homo sapiens olfactory receptor, family 51, subfamily B, member 6 (OR51B6), mRNA [NM_001004750]	NM_001004750
109	A_32_P34138	0.0008	2.355036	FAM25A	Homo sapiens family with sequence similarity 25, member A (FAM25A), mRNA [NM_001146157]	NM_001146157
110	A_23_P3371	0.0333	2.3534653	RASGRF1	Homo sapiens Ras protein-specific guanine nucleotide-releasing factor 1 (RASGRF1), transcript variant 1, mRNA [NM_002891]	NM_002891
111	A_23_P259442	0.0418	2.3479316	CPE	Homo sapiens carboxypeptidase E (CPE), mRNA [NM_001873]	NM_001873
112	A_33_P3288159	0.0010	2.338584	ASPM	Homo sapiens asp (abnormal spindle) homolog, microcephaly associated (Drosophila) (ASPM), transcript variant 1, mRNA [NM_018136]	NM_018136
113	A_32_P9543	0.0110	2.3349195	APOBEC3A	Homo sapiens apolipoprotein B mRNA editing enzyme, catalytic polypeptide-like 3A (APOBEC3A), transcript variant 1, mRNA [NM_145699]	NM_145699
114	A_23_P126649	0.0407	2.3322053	PGBD5	Homo sapiens piggyBac transposable element derived 5 (PGBD5), mRNA [NM_024554]	NM_024554
115	A_24_P177553	0.0059	2.3271344		AGENCOURT_10615922 NIH_MGC_141 Homo sapiens cDNA clone IMAGE:6744194 5', mRNA sequence [BU963192]	BU963192
116	A_33_P3363465	0.0083	2.3134522		Homo sapiens cDNA FLJ43298 fis, clone NCRRP1000129. [AK125288]	AK125288
117	A_23_P107981	0.0280	2.3118186	SULT2B1	Homo sapiens sulfotransferase family, cytosolic, 2B, member 1 (SULT2B1), transcript variant 1, mRNA [NM_004605]	NM_004605
118	A_19_P00803864	0.0104	2.298462	FAM105A	Homo sapiens family with sequence similarity 105, member A (FAM105A), mRNA [NM_019018]	NM_019018
119	A_33_P3382924	0.0161	2.2916381	SPARC	Homo sapiens secreted protein, acidic, cysteine-rich (osteonectin) (SPARC), mRNA [NM_003118]	NM_003118
120	A_19_P00322152	0.0278	2.2842584	XLOC_l2_008434	BROAD Institute lincRNA (XLOC_l2_008434), lincRNA [TCONS_l2_00016080]	AK091865
121	A_19_P00322259	0.0413	2.278918	LOC100506374	PREDICTED: Homo sapiens hypothetical LOC100506374 (LOC100506374), miscRNA [XR_109498]	XR_109498
122	A_24_P303091	0.0063	2.2695482	CXCL10	Homo sapiens chemokine (C-X-C motif) ligand 10 (CXCL10), mRNA [NM_001565]	NM_001565
123	A_24_P102315	0.0494	2.2592964	KIAA1755	Homo sapiens KIAA1755 (KIAA1755), mRNA [NM_001029864]	NM_001029864
124	A_23_P372478	0.0492	2.2567427	SERPINA9	Homo sapiens serpin peptidase inhibitor, clade A (alpha-1 antiproteinase, antitrypsin), member 9 (SERPINA9), transcript variant A, mRNA [NM_175739]	NM_175739
125	A_33_P3305885	0.0356	2.2439618	WNK2	Homo sapiens WNK lysine deficient protein kinase 2 (WNK2), mRNA [NM_006648]	NM_006648
126	A_33_P3386621	0.0432	2.240534		Homo sapiens, clone IMAGE:5788184, mRNA. [BC046191]	BC046191
127	A_33_P3357397	0.0026	2.2397895		T cell receptor beta variable 29-1 [Source:HGNC Symbol;Acc:12210] [ENST00000422143]	AB306246
128	A_23_P83579	0.0492	2.2137465	ARNT2	Homo sapiens aryl-hydrocarbon receptor nuclear translocator 2 (ARNT2), mRNA [NM_014862]	NM_014862
129	A_33_P3590259	0.0304	2.2062783	CXCL14	Homo sapiens chemokine (C-X-C motif) ligand 14 (CXCL14), mRNA [NM_004887]	NM_004887
130	A_23_P131074	0.0001	2.205123	THEG	Homo sapiens Theg homolog (mouse) (THEG), transcript variant 1, mRNA [NM_016585]	NM_016585
131	A_23_P132515	0.0082	2.202624	SIDT1	Homo sapiens SID1 transmembrane family, member 1 (SIDT1), mRNA [NM_017699]	NM_017699
132	A_33_P3343175	0.0100	2.1811285	CXCL10	Homo sapiens chemokine (C-X-C motif) ligand 10 (CXCL10), mRNA [NM_001565]	NM_001565
133	A_23_P351844	0.0025	2.1808178	CD1B	Homo sapiens CD1b molecule (CD1B), mRNA [NM_001764]	NM_001764
134	A_33_P3410284	0.0159	2.1755111	DOCK9	dedicator of cytokinesis 9 [Source:HGNC Symbol;Acc:14132] [ENST00000472874]	AK090793

135	A_24_P73535	0.0071	2.1729858	C14orf119	Homo sapiens chromosome 14 open reading frame 119 (C14orf119), mRNA [NM_017924]	NM_017924
136	A_23_P16252	0.0331	2.1519809	KLK1	Homo sapiens kallikrein 1 (KLK1), mRNA [NM_002257]	NM_002257
137	A_33_P3209591	0.0310	2.143375	AQP3	Homo sapiens aquaporin 3 (Gill blood group) (AQP3), mRNA [NM_004925]	NM_004925
138	A_23_P257043	0.0092	2.1365895	GEM	Homo sapiens GTP binding protein overexpressed in skeletal muscle (GEM), transcript variant 1, mRNA [NM_005261]	NM_005261
139	A_24_P307599	0.0247	2.1310613	NAG20	PREDICTED: Homo sapiens NAG20 (NAG20), miscRNA [XR_108823]	XR_108823
140	A_23_P103672	0.0276	2.1248627	NES	Homo sapiens nestin (NES), mRNA [NM_006617]	NM_006617
141	A_33_P3406939	0.0190	2.120757	KIF24	Homo sapiens kinesin family member 24 (KIF24), mRNA [NM_194313]	NM_194313
142	A_33_P3241591	0.0076	2.1196752	LOC100131864	Homo sapiens cDNA FLJ36900 fis, clone BRACE2001954. [AK094219]	AK094219
143	A_24_P298587	0.0357	2.1182506		Putative transmembrane protein C8orfK29 [Source:UniProtKB/Swiss-Prot;Acc:Q2WGJ8] [ENST00000531225]	AB196634
144	A_23_P397208	0.0241	2.1114416	GSTM2	Homo sapiens glutathione S-transferase mu 2 (muscle) (GSTM2), transcript variant 1, mRNA [NM_000848]	NM_000848
145	A_33_P3398712	0.0309	2.1106145		DB201663 TRACH2 Homo sapiens cDNA clone TRACH2011388 5', mRNA sequence [DB201663]	DB201663
146	A_23_P5258	0.0462	2.1003683	OR10H4	Homo sapiens olfactory receptor, family 10, subfamily H, member 4 (OR10H4), mRNA [NM_001004465]	NM_001004465
147	A_24_P481824	0.0010	2.0974214	DPY19L4	Homo sapiens dpy-19-like 4 (C. elegans) (DPY19L4), mRNA [NM_181787]	NM_181787
148	A_32_P83049	0.0011	2.094928	EFR3B	Homo sapiens EFR3 homolog B (S. cerevisiae) (EFR3B), mRNA [NM_014971]	NM_014971
149	A_33_P3394404	0.0155	2.0937936	TSPAN32	Homo sapiens tetraspanin 32 (TSPAN32), mRNA [NM_139022]	NM_139022
150	A_33_P3215392	0.0040	2.0929492	EXOC3L2	Homo sapiens exocyst complex component 3-like 2 (EXOC3L2), mRNA [NM_138568]	NM_138568
151	A_33_P3313801	0.0202	2.0921676	CCDC34	Homo sapiens coiled-coil domain containing 34 (CCDC34), transcript variant 2, mRNA [NM_080654]	NM_080654
152	A_23_P131789	0.0209	2.0812764	BPI	Homo sapiens bactericidal/permeability-increasing protein (BPI), mRNA [NM_001725]	NM_001725
153	A_23_P166400	0.0174	2.0780158	RASL10A	Homo sapiens RAS-like, family 10, member A (RASL10A), transcript variant 2, mRNA [NM_001007279]	NM_001007279
154	A_23_P51085	0.0130	2.0701516	SPC25	Homo sapiens SPC25, NDC80 kinetochore complex component, homolog (S. cerevisiae) (SPC25), mRNA [NM_020675]	NM_020675
155	A_24_P365975	0.0294	2.0628846	COL8A2	Homo sapiens collagen, type VIII, alpha 2 (COL8A2), mRNA [NM_005202]	NM_005202
156	A_23_P501722	0.0498	2.0584342	TSPAN32	Homo sapiens tetraspanin 32 (TSPAN32), mRNA [NM_139022]	NM_139022
157	A_23_P31240	0.0365	2.057231	GAL3ST4	Homo sapiens galactose-3-O-sulfotransferase 4 (GAL3ST4), mRNA [NM_024637]	NM_024637
158	A_23_P91512	0.0329	2.0525954	CLDN14	Homo sapiens claudin 14 (CLDN14), transcript variant 1, mRNA [NM_144492]	NM_144492
159	A_23_P161769	0.0018	2.051939	FXYD2	Homo sapiens FXYD domain containing ion transport regulator 2 (FXYD2), transcript variant b, mRNA [NM_021603]	NM_021603
160	A_33_P3412013	0.0412	2.0491457	C9orf29	Homo sapiens chromosome 9 open reading frame 29 (C9orf29), non-coding RNA [NR_034087]	NR_034087
161	A_33_P3387616	0.0019	2.0439055	RHPN1	Homo sapiens rhophilin, Rho GTPase binding protein 1 (RHPN1), mRNA [NM_052924]	NM_052924
162	A_33_P3357007	0.0241	2.0287971	C1QTNF9B	Homo sapiens C1q and tumor necrosis factor related protein 9B (C1QTNF9B), mRNA [NM_001007537]	NM_001007537

163	A_33_P3230254	0.0292	2.0228076	NCAPG	Homo sapiens non-SMC condensin I complex, subunit G (NCAPG), mRNA [NM_022346]	NM_022346
164	A_24_P417352	0.0416	2.0163896		immunoglobulin heavy constant mu [Source:HGNC Symbol;Acc:5541] [ENST00000390559]	BX161420
165	A_24_P321125	0.0041	2.0146863	LRRC18	Homo sapiens leucine rich repeat containing 18 (LRRC18), mRNA [NM_001006939]	NM_001006939
166	A_33_P3323486	0.0376	2.0076714		Synthetic construct DNA, clone: pF1KE0827, Homo sapiens OR4A13P gene for Putative olfactory receptor, family 4, subfamily A, member 13, without stop codon, in Flexi system. [AB529256]	AB529256
167	A_33_P3209541	0.0002	2.0022693	KIAA1875	Homo sapiens KIAA1875 (KIAA1875), non-coding RNA [NR_024207]	NR_024207
168	A_23_P354341	0.0029	2.0010567	CD160	Homo sapiens CD160 molecule (CD160), mRNA [NM_007053]	NM_007053

The probes that represent the genes differentially down-regulated in the macrophages cultured with iRBCs compared with those in the macrophages cultured with RBCs were determined by using a moderated T-test. The application of the moderated T-test ($P < 0.05$, fold change > 2.0) led us to identify 237 probes that represent 168 annotated genes in the macrophages cultured with iRBCs.

[a]Fold change in down-regulation indicates the mean expression level of the gene in macrophages co-cultured with iRBCs normalized to that in macrophages co-cultured with RBCs.

Supplementary Table S2. Genes significantly differentially up-regulated in macrophages phagocytizing iRBCs compared with those in macrophages phagocytizing RBCs.

#	Probe Name	*P* value	Fold change[a]	Gene Symbol	Description	Genbank Accession
1	A_33_P3211968	0.012	15.439		PREDICTED: Homo sapiens hypothetical LOC100133207 (LOC100133207), miscRNA [XR_110242]	XR_110242
2	A_24_P555066	0.044	4.613	SMTNL2	Homo sapiens smoothelin-like 2 (SMTNL2), transcript variant 2, mRNA [NM_198501]	NM_198501
3	A_33_P3323902	0.042	4.569	DEFB130	Homo sapiens defensin, beta 130 (DEFB130), mRNA [NM_001037804]	NM_001037804
4	A_33_P3416588	0.001	4.543	RIT2	Homo sapiens Ras-like without CAAX 2 (RIT2), mRNA [NM_002930]	NM_002930
5	A_24_P122337	0.010	4.459	SYTL4	Homo sapiens synaptotagmin-like 4 (SYTL4), transcript variant 1, mRNA [NM_080737]	NM_080737
6	A_23_P163087	0.004	4.198	NID2	Homo sapiens nidogen 2 (osteonidogen) (NID2), mRNA [NM_007361]	NM_007361
7	A_23_P58009	0.001	4.049	C3orf52	Homo sapiens chromosome 3 open reading frame 52 (C3orf52), transcript variant 2, mRNA [NM_024616]	NM_024616
8	A_23_P320290	0.000	3.967	ZNF827	Homo sapiens zinc finger protein 827 (ZNF827), mRNA [NM_178835]	NM_178835
9	A_33_P3416634	0.010	3.912	PRSS41	Homo sapiens protease, serine, 41 (PRSS41), mRNA [NM_001135086]	NM_001135086
10	A_23_P126248	0.003	3.502	RNF186	Homo sapiens ring finger protein 186 (RNF186), mRNA [NM_019062]	NM_019062
11	A_19_P00805212	0.002	3.382	C6orf147	Homo sapiens chromosome 6 open reading frame 147 (C6orf147), non-coding RNA [NR_027005]	NR_027005
12	A_23_P127915	0.000	3.360	STK33	Homo sapiens serine/threonine kinase 33 (STK33), mRNA [NM_030906]	NM_030906
13	A_24_P330822	0.000	3.316	HNF1B	Homo sapiens HNF1 homeobox B (HNF1B), transcript variant 1, mRNA [NM_000458]	NM_000458
14	A_33_P3273474	0.038	3.313	CD1C	Homo sapiens CD1c molecule (CD1C), mRNA [NM_001765]	NM_001765
15	A_23_P80503	0.000	3.242	ROBO1	Homo sapiens roundabout, axon guidance receptor, homolog 1 (Drosophila) (ROBO1), transcript variant 2, mRNA [NM_133631]	NM_133631
16	A_33_P3469658	0.002	3.218	LOC284570	Homo sapiens, clone IMAGE:4941949, mRNA. [BC040156]	BC040156
17	A_23_P129856	0.001	3.191	HIC1	Homo sapiens hypermethylated in cancer 1 (HIC1), transcript variant 1, mRNA [NM_006497]	NM_006497
18	A_23_P389897	0.001	3.153	NGFR	Homo sapiens nerve growth factor receptor (NGFR), mRNA [NM_002507]	NM_002507
19	A_33_P3305763	0.000	3.104		Homo sapiens chromosome 8 open reading frame 6, mRNA (cDNA clone IMAGE:7262516). [BC069683]	BC069683
20	A_32_P504827	0.008	3.095	MUC12	Homo sapiens mucin 12, cell surface associated (MUC12), mRNA [NM_001164462]	NM_001164462
21	A_33_P3264238	0.018	3.053	ZNF280B	Homo sapiens zinc finger protein 280B (ZNF280B), mRNA [NM_080764]	NM_080764
22	A_33_P3259615	0.003	3.042	TTC36	Homo sapiens tetratricopeptide repeat domain 36 (TTC36), mRNA [NM_001080441]	NM_001080441
23	A_19_P00315804	0.002	3.038	FLJ35024	Homo sapiens uncharacterized LOC401491 (FLJ35024), non-coding RNA [NR_015375]	NR_015375
24	A_33_P3222630	0.003	2.948	FBXO43	Homo sapiens F-box protein 43 (FBXO43), transcript variant 2, mRNA [NM_001029860]	NM_001029860

25	A_33_P3440636	0.022	2.946	LOC286071	Homo sapiens cDNA FLJ34440 fis, clone HLUNG2001214. [AK091759]	AK091759
26	A_33_P3289701	0.000	2.939	OR2T12	Homo sapiens olfactory receptor, family 2, subfamily T, member 12 (OR2T12), mRNA [NM_001004692]	NM_001004692
27	A_33_P3333282	0.003	2.896	FGF11	Homo sapiens fibroblast growth factor 11 (FGF11), mRNA [NM_004112]	NM_004112
28	A_33_P3519424	0.005	2.880	LOC386597	Homo sapiens cDNA FLJ32573 fis, clone SPLEN2000210. [AK057135]	AK057135
29	A_33_P3365646	0.016	2.868	LOC100128437	Homo sapiens cDNA FLJ44089 fis, clone TESTI4041624. [AK126077]	AK126077
30	A_24_P331830	0.009	2.842	KAZN	Homo sapiens kazrin, periplakin interacting protein (KAZN), transcript variant A, mRNA [NM_015209]	NM_015209
31	A_23_P104762	0.000	2.825	YAP1	Homo sapiens Yes-associated protein 1 (YAP1), transcript variant 2, mRNA [NM_006106]	NM_006106
32	A_19_P00321765	0.034	2.820	LOC100287765	Homo sapiens uncharacterized LOC100287765 (LOC100287765), non-coding RNA [NR_038988]	NR_038988
33	A_33_P3309206	0.003	2.812	GABRB3	gamma-aminobutyric acid (GABA) A receptor, beta 3 [Source:HGNC Symbol;Acc:4083] [ENST00000556166]	BC038837
34	A_23_P162579	0.006	2.795	HSPB8	Homo sapiens heat shock 22kDa protein 8 (HSPB8), mRNA [NM_014365]	NM_014365
35	A_23_P253301	0.024	2.793	PFN2	Homo sapiens profilin 2 (PFN2), transcript variant 1, mRNA [NM_053024]	NM_053024
36	A_23_P39525	0.000	2.761	FAM124B	Homo sapiens family with sequence similarity 124B (FAM124B), transcript variant 2, mRNA [NM_024785]	NM_024785
37	A_19_P00321687	0.025	2.760	LOC92249	Homo sapiens uncharacterized LOC92249 (LOC92249), non-coding RNA [NR_015353]	NR_015353
38	A_33_P3409392	0.031	2.756	FZD6	Homo sapiens frizzled family receptor 6 (FZD6), transcript variant 1, mRNA [NM_003506]	NM_003506
39	A_33_P3246017	0.000	2.747	CTAGE5	CTAGE family, member 5 [Source:HGNC Symbol;Acc:7057] [ENST00000396165]	AK091252
40	A_23_P160751	0.028	2.727	FCRL2	Homo sapiens Fc receptor-like 2 (FCRL2), mRNA [NM_030764]	NM_030764
41	A_23_P101131	0.006	2.726	GRP	Homo sapiens gastrin-releasing peptide (GRP), transcript variant 1, mRNA [NM_002091]	NM_002091
42	A_23_P423074	0.004	2.719	FAM169A	Homo sapiens family with sequence similarity 169, member A (FAM169A), mRNA [NM_015566]	NM_015566
43	A_33_P3406886	0.028	2.709	LINC00478	Homo sapiens long intergenic non-protein coding RNA 478 (LINC00478), transcript variant 1, non-coding RNA [NR_027790]	NR_027790
44	A_33_P3259135	0.034	2.709	D4S234E	Homo sapiens DNA segment on chromosome 4 (unique) 234 expressed sequence (D4S234E), transcript variant 1, mRNA [NM_014392]	NM_014392
45	A_33_P3405888	0.004	2.699	LOC100133130	Homo sapiens clone FLB4246 PRO1102 mRNA, complete cds. [AF130105]	AF130105
46	A_33_P3316233	0.001	2.694	MRPS18C	Homo sapiens mitochondrial ribosomal protein S18C (MRPS18C), nuclear gene encoding mitochondrial protein, mRNA [NM_016067]	NM_016067
47	A_33_P3355937	0.046	2.692	LOC284276	Homo sapiens uncharacterized LOC284276 (LOC284276), non-coding RNA [NR_015417]	NR_015417
48	A_33_P3416757	0.002	2.688	PRLR	Homo sapiens prolactin receptor (PRLR), transcript variant 2, mRNA [NM_001204315]	NM_001204315
49	A_33_P3214914	0.003	2.657	MUC22	Homo sapiens mucin 22 (MUC22), mRNA [NM_001198815]	NM_001198815
50	A_33_P3414689	0.005	2.649		Homo sapiens FP15529 mRNA, complete cds. [AF495723]	AF495723
51	A_33_P3353345	0.003	2.646	SULT4A1	Homo sapiens sulfotransferase family 4A, member 1	NM_014351

					(SULT4A1), mRNA [NM_014351]	
52	A_33_P3303146	0.046	2.645	ATG4C	Homo sapiens ATG4 autophagy related 4 homolog C (S. cerevisiae) (ATG4C), transcript variant 1, mRNA [NM_032852]	NM_032852
53	A_33_P3274084	0.018	2.642	RHBDL3	Homo sapiens rhomboid, veinlet-like 3 (Drosophila) (RHBDL3), mRNA [NM_138328]	NM_138328
54	A_33_P3240273	0.002	2.627		family with sequence similarity 95, member B1 [Source:HGNC Symbol;Acc:32318] [ENST00000455995]	XR_133518
55	A_24_P365506	0.003	2.616	FERMT1	Homo sapiens fermitin family member 1 (FERMT1), mRNA [NM_017671]	NM_017671
56	A_23_P358709	0.006	2.596	AHRR	Homo sapiens aryl-hydrocarbon receptor repressor (AHRR), transcript variant 1, mRNA [NM_020731]	NM_020731
57	A_33_P3289621	0.001	2.589	LOC283143	Homo sapiens cDNA FLJ33283 fis, clone ASTRO2009177. [AK090602]	AK090602
58	A_33_P3415340	0.046	2.586	ALS2	amyotrophic lateral sclerosis 2 (juvenile) [Source:HGNC Symbol;Acc:443] [ENST00000410052]	AK090992
59	A_23_P215634	0.000	2.582	IGFBP3	Homo sapiens insulin-like growth factor binding protein 3 (IGFBP3), transcript variant 1, mRNA [NM_001013398]	NM_001013398
60	A_23_P119763	0.000	2.569	ABCG5	Homo sapiens ATP-binding cassette, sub-family G (WHITE), member 5 (ABCG5), mRNA [NM_022436]	NM_022436
61	A_33_P3299560	0.019	2.568	LEKR1	Homo sapiens cDNA FLJ37161 fis, clone BRACE2026725. [AK094480]	AK094480
62	A_33_P3399253	0.000	2.561		MGC13nov.3.1.L1.1.G04.F.1 NIH_MGC_331 Homo sapiens cDNA clone MGC13nov.3.1.L1.1.G04, mRNA sequence [EG328730]	EG328730
63	A_33_P3235701	0.021	2.552	ZCCHC11	zinc finger, CCHC domain containing 11 [Source:HGNC Symbol;Acc:28981] [ENST00000371541]	BX648783
64	A_33_P3306110	0.044	2.528	CALCRL	Homo sapiens calcitonin receptor-like (CALCRL), mRNA [NM_005795]	NM_005795
65	A_24_P21829	0.024	2.508	SLC22A24	Homo sapiens solute carrier family 22, member 24 (SLC22A24), mRNA [NM_001136506]	NM_001136506
66	A_33_P3395605	0.044	2.506	TMEM119	Homo sapiens transmembrane protein 119 (TMEM119), mRNA [NM_181724]	NM_181724
67	A_23_P6818	0.024	2.503	SEMA3G	Homo sapiens sema domain, immunoglobulin domain (Ig), short basic domain, secreted, (semaphorin) 3G (SEMA3G), mRNA [NM_020163]	NM_020163
68	A_32_P96000	0.034	2.501	ETV3L	Homo sapiens ets variant 3-like (ETV3L), mRNA [NM_001004341]	NM_001004341
69	A_23_P154972	0.000	2.500	ZNF280A	Homo sapiens zinc finger protein 280A (ZNF280A), mRNA [NM_080740]	NM_080740
70	A_23_P252306	0.001	2.487	ID1	Homo sapiens inhibitor of DNA binding 1, dominant negative helix-loop-helix protein (ID1), transcript variant 1, mRNA [NM_002165]	NM_002165
71	A_23_P27107	0.012	2.482	TM4SF5	Homo sapiens transmembrane 4 L six family member 5 (TM4SF5), mRNA [NM_003963]	NM_003963
72	A_32_P14762	0.023	2.474	OOEP	Homo sapiens oocyte expressed protein homolog (dog) (OOEP), mRNA [NM_001080507]	NM_001080507
73	A_23_P111995	0.023	2.470	LOXL2	Homo sapiens lysyl oxidase-like 2 (LOXL2), mRNA [NM_002318]	NM_002318
74	A_33_P3219527	0.001	2.469	RTKN2	rhotekin 2 [Source:HGNC Symbol;Acc:19364] [ENST00000395265]	AK096671
75	A_23_P399797	0.004	2.462	SMAD5-AS1	Homo sapiens SMAD5 antisense RNA 1 (non-protein coding) (SMAD5-AS1), non-coding RNA [NR_026763]	NR_026763
76	A_23_P304682	0.019	2.456	EMP2	Homo sapiens epithelial membrane protein 2 (EMP2), mRNA [NM_001424]	NM_001424
77	A_33_P3298120	0.045	2.444	HEPN1	Homo sapiens hepatocellular carcinoma, down-regulated 1	NM_001037558

					(HEPN1), mRNA [NM_001037558]	
78	A_24_P489164	0.002	2.442	LOC100131943	Homo sapiens clone DNA93013 RVLA1944 (UNQ1944) mRNA, complete cds. [AY358202]	AY358202
79	A_24_P320545	0.009	2.437	PTK7	Homo sapiens PTK7 protein tyrosine kinase 7 (PTK7), transcript variant PTK7-1, mRNA [NM_002821]	NM_002821
80	A_23_P59285	0.013	2.433	GCM2	Homo sapiens glial cells missing homolog 2 (Drosophila) (GCM2), mRNA [NM_004752]	NM_004752
81	A_19_P00321575	0.013	2.428	BPESC1	Homo sapiens blepharophimosis, epicanthus inversus and ptosis, candidate 1 (non-protein coding) (BPESC1), non-coding RNA [NR_026783]	NR_026783
82	A_24_P6517	0.031	2.423	PLEKHG1	Homo sapiens pleckstrin homology domain containing, family G (with RhoGef domain) member 1 (PLEKHG1), mRNA [NM_001029884]	NM_001029884
83	A_33_P3230414	0.007	2.420	ZNF774	Homo sapiens zinc finger protein 774 (ZNF774), mRNA [NM_001004309]	NM_001004309
84	A_33_P3212072	0.048	2.416	GORAB	Homo sapiens golgin, RAB6-interacting (GORAB), transcript variant 3, mRNA [NM_001146039]	NM_001146039
85	A_33_P3331446	0.048	2.405	SERP1	stress-associated endoplasmic reticulum protein 1 [Source:HGNC Symbol;Acc:10759] [ENST00000479209]	AK125413
86	A_23_P164451	0.022	2.401	TBX2	Homo sapiens T-box 2 (TBX2), mRNA [NM_005994]	NM_005994
87	A_23_P114057	0.011	2.397	SEMA4C	Homo sapiens sema domain, immunoglobulin domain (Ig), transmembrane domain (TM) and short cytoplasmic domain, (semaphorin) 4C (SEMA4C), mRNA [NM_017789]	NM_017789
88	A_23_P12643	0.001	2.396	AS3MT	Homo sapiens arsenic (+3 oxidation state) methyltransferase (AS3MT), mRNA [NM_020682]	NM_020682
89	A_33_P3310189	0.006	2.395	ADRB1	Homo sapiens adrenergic, beta-1-, receptor (ADRB1), mRNA [NM_000684]	NM_000684
90	A_19_P00321061	0.013	2.392	LOC100499221	ALU8_HUMAN (P39195) Alu subfamily SX sequence contamination warning entry, partial (10%) [THC2486640]	AK127052
91	A_33_P3239455	0.004	2.389	GTF2IRD2	Homo sapiens GTF2I repeat domain containing 2 (GTF2IRD2), mRNA [NM_173537]	NM_173537
92	A_32_P65616	0.011	2.384	PRL	Homo sapiens prolactin (PRL), transcript variant 1, mRNA [NM_000948]	NM_000948
93	A_33_P3358213	0.007	2.382	PADI6	Homo sapiens peptidyl arginine deiminase, type VI (PADI6), mRNA [NM_207421]	NM_207421
94	A_33_P3402817	0.027	2.379	CRHR1	Homo sapiens corticotropin releasing hormone receptor 1 (CRHR1), transcript variant 4, mRNA [NM_001145148]	NM_001145148
95	A_33_P3241782	0.014	2.375	ADD2	Homo sapiens adducin 2 (beta) (ADD2), transcript variant 1, mRNA [NM_001617]	NM_001617
96	A_33_P3289025	0.032	2.370	FAM176A	Homo sapiens family with sequence similarity 176, member A (FAM176A), transcript variant 1, mRNA [NM_001135032]	NM_001135032
97	A_33_P3329088	0.004	2.362	PRSS8	Homo sapiens protease, serine, 8 (PRSS8), mRNA [NM_002773]	NM_002773
98	A_23_P19663	0.015	2.353	CTGF	Homo sapiens connective tissue growth factor (CTGF), mRNA [NM_001901]	NM_001901
99	A_33_P3268989	0.034	2.347		ASCC1_HUMAN (Q8N9N2) Activating signal cointegrator 1 complex subunit 1 (ASC-1 complex subunit p50) (Trip4 complex subunit p50), complete [THC2581013]	THC2581013
100	A_33_P3235247	0.037	2.346	LOC100130579	PREDICTED: Homo sapiens hypothetical LOC100130579 (LOC100130579), miscRNA [XR_109158]	XR_109158
101	A_23_P500353	0.017	2.341	KCNN2	Homo sapiens potassium intermediate/small conductance	NM_021614

					calcium-activated channel, subfamily N, member 2 (KCNN2), transcript variant 1, mRNA [NM_021614]	
102	A_33_P3286873	0.017	2.338	CD247	CD247 molecule [Source:HGNC Symbol;Acc:1677] [ENST00000483825]	AK128376
103	A_33_P3281003	0.049	2.330		Homo sapiens cDNA FLJ45587 fis, clone BRTHA3014105. [AK127494]	AK127494
104	A_33_P3725324	0.003	2.325	USP9Y	Homo sapiens ubiquitin specific peptidase 9, Y-linked (USP9Y), mRNA [NM_004654]	NM_004654
105	A_33_P3280646	0.007	2.320	CASR	Homo sapiens calcium-sensing receptor (CASR), transcript variant 1, mRNA [NM_001178065]	NM_001178065
106	A_24_P192627	0.021	2.314	MLLT3	Homo sapiens myeloid/lymphoid or mixed-lineage leukemia (trithorax homolog, Drosophila); translocated to, 3 (MLLT3), mRNA [NM_004529]	NM_004529
107	A_23_P44244	0.029	2.314	SMARCA1	Homo sapiens SWI/SNF related, matrix associated, actin dependent regulator of chromatin, subfamily a, member 1 (SMARCA1), transcript variant 1, mRNA [NM_003069]	NM_003069
108	A_33_P3832680	0.012	2.306	FAM25C	Homo sapiens cDNA clone IMAGE:40133024. [BC127791]	BC127791
109	A_32_P222695	0.009	2.298	ARHGEF37	Homo sapiens Rho guanine nucleotide exchange factor (GEF) 37 (ARHGEF37), mRNA [NM_001001669]	NM_001001669
110	A_33_P3290303	0.003	2.294	LOC100128278	PREDICTED: Homo sapiens hypothetical LOC100128278 (LOC100128278), miscRNA [XR_110306]	XR_110306
111	A_32_P231493	0.025	2.292		Homo sapiens clone IMAGE:1257951, mRNA sequence. [AF339771]	AF339771
112	A_24_P269062	0.011	2.291	SPRY4	Homo sapiens sprouty homolog 4 (Drosophila) (SPRY4), transcript variant 1, mRNA [NM_030964]	NM_030964
113	A_33_P3324383	0.020	2.287	FIGNL2	Homo sapiens fidgetin-like 2 (FIGNL2), mRNA [NM_001013690]	NM_001013690
114	A_33_P3318971	0.008	2.283	METTL20	methyltransferase like 20 [Source:HGNC Symbol;Acc:28739] [ENST00000357721]	AL832339
115	A_33_P3259112	0.029	2.282	FLJ41278	Homo sapiens uncharacterized LOC400046 (FLJ41278), non-coding RNA [NR_033988]	NR_033988
116	A_33_P3375299	0.006	2.282		Uncharacterized protein [Source:UniProtKB/TrEMBL;Acc:B5MCS7] [ENST00000431083]	BF244550
117	A_23_P259692	0.001	2.280	PSAT1	Homo sapiens phosphoserine aminotransferase 1 (PSAT1), transcript variant 1, mRNA [NM_058179]	NM_058179
118	A_23_P108673	0.010	2.279	FAM176A	Homo sapiens family with sequence similarity 176, member A (FAM176A), transcript variant 2, mRNA [NM_032181]	NM_032181
119	A_32_P59010	0.009	2.278	TNFAIP8L3	Homo sapiens tumor necrosis factor, alpha-induced protein 8-like 3 (TNFAIP8L3), mRNA [NM_207381]	NM_207381
120	A_33_P3241145	0.045	2.272		Homo sapiens cDNA clone IMAGE:5273698. [BC047414]	BC047414
121	A_24_P301186	0.017	2.271	WDR89	Homo sapiens WD repeat domain 89 (WDR89), transcript variant 1, mRNA [NM_001008726]	NM_001008726
122	A_19_P00318418	0.000	2.269	TBXAS1	Homo sapiens thromboxane A synthase 1 (platelet) (TBXAS1), transcript variant 5, mRNA [NM_001166254]	NM_001166254
123	A_23_P376704	0.002	2.266	CIDEA	Homo sapiens cell death-inducing DFFA-like effector a (CIDEA), transcript variant 1, mRNA [NM_001279]	NM_001279
124	A_23_P64932	0.004	2.266	RIC8B	Homo sapiens resistance to inhibitors of cholinesterase 8 homolog B (C. elegans) (RIC8B), mRNA [NM_018157]	NM_018157
125	A_23_P28186	0.028	2.265	SRD5A2	Homo sapiens steroid-5-alpha-reductase, alpha polypeptide 2 (3-oxo-5 alpha-steroid delta 4-dehydrogenase alpha 2) (SRD5A2), mRNA	NM_000348

					[NM_000348]	
126	A_32_P52519	0.003	2.264	OTOA	Homo sapiens otoancorin (OTOA), transcript variant 2, mRNA [NM_170664]	NM_170664
127	A_33_P3789327	0.011	2.259	SNORD12B	AV755695 BM Homo sapiens cDNA clone BMFBHD10 5', mRNA sequence [AV755695]	AV755695
128	A_33_P3331188	0.005	2.250	ARHGAP23	Homo sapiens Rho GTPase activating protein 23 (ARHGAP23), mRNA [NM_001199417]	NM_001199417
129	A_24_P269779	0.002	2.248	CHST3	Homo sapiens carbohydrate (chondroitin 6) sulfotransferase 3 (CHST3), mRNA [NM_004273]	NM_004273
130	A_24_P207995	0.012	2.244	L1CAM	Homo sapiens L1 cell adhesion molecule (L1CAM), transcript variant 1, mRNA [NM_000425]	NM_000425
131	A_23_P143178	0.034	2.235	LBP	Homo sapiens lipopolysaccharide binding protein (LBP), mRNA [NM_004139]	NM_004139
132	A_33_P3278510	0.002	2.230		Homo sapiens cDNA clone IMAGE:4817893. [BC040680]	BC040680
133	A_23_P42684	0.010	2.226	FLJ20712	PREDICTED: Homo sapiens hypothetical FLJ20712 (FLJ20712), miscRNA [XR_108766]	XR_108766
134	A_33_P3356846	0.042	2.220	MTX3	Homo sapiens metaxin 3 (MTX3), transcript variant 2, mRNA [NM_001010891]	NM_001010891
135	A_23_P142345	0.009	2.219	PRTN3	Homo sapiens proteinase 3 (PRTN3), mRNA [NM_002777]	NM_002777
136	A_23_P331049	0.016	2.215	DPYSL4	Homo sapiens dihydropyrimidinase-like 4 (DPYSL4), mRNA [NM_006426]	NM_006426
137	A_32_P14721	0.038	2.212	DNAH12	Homo sapiens dynein, axonemal, heavy chain 12 (DNAH12), transcript variant 1, mRNA [NM_178504]	NM_178504
138	A_23_P81369	0.033	2.209	ADAM19	Homo sapiens ADAM metallopeptidase domain 19 (ADAM19), mRNA [NM_033274]	NM_033274
139	A_23_P36187	0.020	2.199	SYT8	Homo sapiens synaptotagmin VIII (SYT8), mRNA [NM_138567]	NM_138567
140	A_32_P420520	0.030	2.198	THAP7-AS1	Homo sapiens THAP7 antisense RNA 1 (non-protein coding) (THAP7-AS1), transcript variant 1, non-coding RNA [NR_027051]	NR_027051
141	A_33_P3342056	0.042	2.197	TIGIT	Homo sapiens T cell immunoreceptor with Ig and ITIM domains (TIGIT), mRNA [NM_173799]	NM_173799
142	A_23_P419641	0.040	2.197	DLL4	Homo sapiens delta-like 4 (Drosophila) (DLL4), mRNA [NM_019074]	NM_019074
143	A_33_P3381393	0.046	2.184	VAX1	Homo sapiens ventral anterior homeobox 1 (VAX1), transcript variant 1, mRNA [NM_001112704]	NM_001112704
144	A_33_P3295917	0.023	2.176	IL7	interleukin 7 [Source:HGNC Symbol;Acc:6023] [ENST00000379113]	AB102885
145	A_33_P3300600	0.004	2.176	OR7G1	Homo sapiens olfactory receptor, family 7, subfamily G, member 1 (OR7G1), mRNA [NM_001005192]	NM_001005192
146	A_33_P3258923	0.022	2.175	MKRN3	Homo sapiens makorin ring finger protein 3 (MKRN3), mRNA [NM_005664]	NM_005664
147	A_33_P3295313	0.000	2.174	MS4A2	Homo sapiens membrane-spanning 4-domains, subfamily A, member 2 (Fc fragment of IgE, high affinity I, receptor for; beta polypeptide) (MS4A2), mRNA [NM_000139]	NM_000139
148	A_33_P3319957	0.000	2.173		spermatogenesis associated 1 [Source:HGNC Symbol;Acc:14682] [ENST00000370638]	BC064144
149	A_33_P3358541	0.014	2.168		Homo sapiens cDNA FLJ40473 fis, clone TESTI2042806. [AK097792]	AK097792
150	A_23_P125618	0.011	2.168	GABRA3	Homo sapiens gamma-aminobutyric acid (GABA) A receptor, alpha 3 (GABRA3), mRNA [NM_000808]	NM_000808
151	A_23_P85783	0.001	2.167	PHGDH	Homo sapiens phosphoglycerate dehydrogenase (PHGDH), mRNA [NM_006623]	NM_006623
152	A_19_P00322757	0.005	2.166	MCF2L	PREDICTED: Homo sapiens hypothetical	XR_110307

					LOC100506063 (LOC100506063), miscRNA [XR_110307]	
153	A_23_P55400	0.001	2.165	KRT36	Homo sapiens keratin 36 (KRT36), mRNA [NM_003771]	NM_003771
154	A_33_P3372840	0.017	2.164	CXCL12	Homo sapiens chemokine (C-X-C motif) ligand 12 (CXCL12), transcript variant 3, mRNA [NM_001033886]	NM_001033886
155	A_33_P3664161	0.014	2.160	LOC388946	Homo sapiens transmembrane protein ENSP00000343375 (LOC388946), mRNA [NM_001145051]	NM_001145051
156	A_33_P3402354	0.016	2.160	YLPM1	YLP motif containing 1 [Source:HGNC Symbol;Acc:17798] [ENST00000238571]	L40403
157	A_33_P3374076	0.010	2.157	SEZ6	Homo sapiens seizure related 6 homolog (mouse) (SEZ6), transcript variant 2, mRNA [NM_001098635]	NM_001098635
158	A_33_P3230259	0.021	2.156	NCAPH	Homo sapiens non-SMC condensin I complex, subunit H (NCAPH), mRNA [NM_015341]	NM_015341
159	A_33_P3377187	0.015	2.154	BK250D10.8	Homo sapiens uncharacterized LOC339674 (BK250D10.8), non-coding RNA [NR_024355]	NR_024355
160	A_24_P79040	0.030	2.150	CAPN12	Homo sapiens calpain 12 (CAPN12), mRNA [NM_144691]	NM_144691
161	A_32_P430695	0.014	2.147	C3orf35	Homo sapiens chromosome 3 open reading frame 35 (C3orf35), transcript variant B, mRNA [NM_178339]	NM_178339
162	A_23_P304897	0.011	2.141	BDKRB2	Homo sapiens bradykinin receptor B2 (BDKRB2), mRNA [NM_000623]	NM_000623
163	A_23_P205867	0.044	2.141	NR2E3	Homo sapiens nuclear receptor subfamily 2, group E, member 3 (NR2E3), transcript variant 2, mRNA [NM_014249]	NM_014249
164	A_33_P3229375	0.002	2.134	PSMB11	Homo sapiens proteasome (prosome, macropain) subunit, beta type, 11 (PSMB11), mRNA [NM_001099780]	NM_001099780
165	A_23_P98121	0.002	2.130	FXYD4	Homo sapiens FXYD domain containing ion transport regulator 4 (FXYD4), transcript variant 1, mRNA [NM_173160]	NM_173160
166	A_24_P176714	0.001	2.128	B9D1	Homo sapiens B9 protein domain 1 (B9D1), transcript variant 2, mRNA [NM_015681]	NM_015681
167	A_33_P3318581	0.013	2.122	PLOD2	Homo sapiens procollagen-lysine, 2-oxoglutarate 5-dioxygenase 2 (PLOD2), transcript variant 1, mRNA [NM_182943]	NM_182943
168	A_23_P36018	0.028	2.119	VSIG2	Homo sapiens V-set and immunoglobulin domain containing 2 (VSIG2), mRNA [NM_014312]	NM_014312
169	A_33_P3224495	0.011	2.111		KCNQ5 intronic transcript 1 (non-protein coding) [Source:HGNC Symbol;Acc:41354] [ENST00000445310]	XM_003118553
170	A_33_P3300132	0.008	2.104	FP6628	PREDICTED: Homo sapiens hypothetical LOC100132183 (FP6628), miscRNA [XR_108867]	XR_108867
171	A_32_P161762	0.001	2.104	RUNX2	Homo sapiens runt-related transcription factor 2 (RUNX2), transcript variant 3, mRNA [NM_004348]	NM_004348
172	A_32_P180315	0.026	2.100	C9orf174	Homo sapiens chromosome 9 open reading frame 174 (C9orf174), mRNA [NM_020893]	NM_020893
173	A_23_P385835	0.047	2.098	GPR27	Homo sapiens G protein-coupled receptor 27 (GPR27), mRNA [NM_018971]	NM_018971
174	A_24_P235266	0.000	2.097	GRB10	Homo sapiens growth factor receptor-bound protein 10 (GRB10), transcript variant 4, mRNA [NM_001001555]	NM_001001555
175	A_33_P3461039	0.001	2.093	LOC728705	Homo sapiens cDNA FLJ31150 fis, clone IMR322001534. [AK055712]	AK055712
176	A_32_P76720	0.019	2.091	NT5DC3	Homo sapiens 5'-nucleotidase domain containing 3 (NT5DC3), mRNA [NM_001031701]	NM_001031701
177	A_33_P3318653	0.007	2.090	OR5H6	Homo sapiens olfactory receptor, family 5, subfamily H, member 6 (OR5H6), mRNA [NM_001005479]	NM_001005479
178	A_33_P3400057	0.033	2.090	TCEANC	Homo sapiens transcription elongation factor A (SII) N-terminal and central domain containing (TCEANC),	NM_152634

					mRNA [NM_152634]	
179	A_32_P60065	0.001	2.090	F2RL2	Homo sapiens coagulation factor II (thrombin) receptor-like 2 (F2RL2), mRNA [NM_004101]	NM_004101
180	A_24_P93309	0.006	2.090	CDNF	Homo sapiens cerebral dopamine neurotrophic factor (CDNF), mRNA [NM_001029954]	NM_001029954
181	A_23_P122863	0.000	2.088	GRB10	Homo sapiens growth factor receptor-bound protein 10 (GRB10), transcript variant 4, mRNA [NM_001001555]	NM_001001555
182	A_23_P83976	0.004	2.088	CEP112	Homo sapiens centrosomal protein 112kDa (CEP112), transcript variant 1, mRNA [NM_145036]	NM_145036
183	A_23_P66732	0.007	2.079	GSG2	Homo sapiens germ cell associated 2 (haspin) (GSG2), mRNA [NM_031965]	NM_031965
184	A_33_P3311245	0.045	2.075	KRTAP19-7	Homo sapiens keratin associated protein 19-7 (KRTAP19-7), mRNA [NM_181614]	NM_181614
185	A_33_P3352253	0.023	2.072	MTUS1	Homo sapiens microtubule associated tumor suppressor 1 (MTUS1), transcript variant 2, mRNA [NM_001001925]	NM_001001925
186	A_24_P357266	0.015	2.069	GRPR	Homo sapiens gastrin-releasing peptide receptor (GRPR), mRNA [NM_005314]	NM_005314
187	A_33_P3260563	0.005	2.062	SOX30	Homo sapiens SRY (sex determining region Y)-box 30 (SOX30), transcript variant 2, mRNA [NM_007017]	NM_007017
188	A_33_P3409234	0.049	2.061		DA399191 BRTHA3 Homo sapiens cDNA clone BRTHA3001578 5', mRNA sequence [DA399191]	DA399191
189	A_33_P3883116	0.029	2.061	LOC253962	Homo sapiens uncharacterized LOC253962 (LOC253962), non-coding RNA [NR_038439]	NR_038439
190	A_33_P3377719	0.003	2.056	LOC100129112	Homo sapiens cDNA FLJ41845 fis, clone NT2RI3003095. [AK123839]	AK123839
191	A_19_P00318588	0.049	2.055	Q4B8L8	Q4B8L8_BURVI (Q4B8L8) Regulatory protein, MarR, partial (9%) [THC2606106]	BC082237
192	A_33_P3316508	0.008	2.053	RUNX2	Homo sapiens runt-related transcription factor 2 (RUNX2), transcript variant 2, mRNA [NM_001015051]	NM_001015051
193	A_24_P160401	0.004	2.052	CDCP1	Homo sapiens CUB domain containing protein 1 (CDCP1), transcript variant 2, mRNA [NM_178181]	NM_178181
194	A_19_P00812924	0.008	2.052	LOC100507165	PREDICTED: Homo sapiens hypothetical LOC100507165 (LOC100507165), miscRNA [XR_110530]	XR_110530
195	A_33_P3728698	0.022	2.050	FLJ45248	Homo sapiens cDNA FLJ45248 fis, clone BRHIP2006819. [AK127183]	AK127183
196	A_33_P3284763	0.031	2.048	DMD	Homo sapiens dystrophin (DMD), transcript variant Dp140b, mRNA [NM_004021]	NM_004021
197	A_33_P3402615	0.004	2.042	SLC6A9	Homo sapiens solute carrier family 6 (neurotransmitter transporter, glycine), member 9 (SLC6A9), transcript variant 2, mRNA [NM_201649]	NM_201649
198	A_33_P3500167	0.025	2.042	ACAN	Homo sapiens aggrecan (ACAN), transcript variant 1, mRNA [NM_001135]	NM_001135
199	A_23_P6909	0.013	2.042	CCRL1	Homo sapiens chemokine (C-C motif) receptor-like 1 (CCRL1), transcript variant 1, mRNA [NM_178445]	NM_178445
200	A_23_P413303	0.011	2.041	DKFZP434I0714	Homo sapiens uncharacterized protein DKFZP434I0714 (DKFZP434I0714), non-coding RNA [NR_033797]	NR_033797
201	A_33_P3293888	0.002	2.039	AFF2	Homo sapiens AF4/FMR2 family, member 2 (AFF2), transcript variant 1, mRNA [NM_002025]	NM_002025
202	A_33_P3406572	0.021	2.033	LOC149773	Homo sapiens cDNA FLJ34385 fis, clone HCHON1000142. [AK091704]	AK091704
203	A_24_P208436	0.008	2.028	PDE1A	Homo sapiens phosphodiesterase 1A, calmodulin-dependent (PDE1A), transcript variant 2, mRNA [NM_001003683]	NM_001003683
204	A_33_P3265494	0.044	2.025	C6orf138	Homo sapiens chromosome 6 open reading frame 138 (C6orf138), transcript variant 1, mRNA [NM_001013732]	NM_001013732

205	A_33_P3217356	0.000	2.024	LOC388630	Homo sapiens UPF0632 protein A (LOC388630), mRNA [NM_001194986]	NM_001194986
206	A_33_P3367112	0.012	2.024		full-length cDNA clone CS0DD005YE10 of Neuroblastoma Cot 50-normalized of Homo sapiens (human) [CR607463]	CR607463
207	A_33_P3280805	0.003	2.024	LMO7	Homo sapiens LIM domain 7 (LMO7), transcript variant 2, mRNA [NM_015842]	NM_015842
208	A_23_P401547	0.033	2.022	PVRL3	Homo sapiens poliovirus receptor-related 3 (PVRL3), transcript variant 1, mRNA [NM_015480]	NM_015480
209	A_33_P3824237	0.033	2.020	LOC439990	Homo sapiens uncharacterized LOC439990 (LOC439990), non-coding RNA [NR_038464]	NR_038464
210	A_23_P141802	0.014	2.019	SERPINB7	Homo sapiens serpin peptidase inhibitor, clade B (ovalbumin), member 7 (SERPINB7), transcript variant 2, mRNA [NM_001040147]	NM_001040147
211	A_33_P3338011	0.040	2.018	ZNF480	Homo sapiens zinc finger protein 480 (ZNF480), mRNA [NM_144684]	NM_144684
212	A_33_P3233436	0.034	2.017	PNMA6C	Homo sapiens paraneoplastic antigen like 6C (PNMA6C), mRNA [NM_001170944]	NM_001170944
213	A_24_P940086	0.002	2.015	NXPH3	Homo sapiens neurexophilin 3 (NXPH3), mRNA [NM_007225]	NM_007225
214	A_33_P3561747	0.020	2.012	C16orf82	Homo sapiens chromosome 16 open reading frame 82 (C16orf82), mRNA [NM_001145545]	NM_001145545
215	A_23_P117896	0.040	2.009	TGM7	Homo sapiens transglutaminase 7 (TGM7), mRNA [NM_052955]	NM_052955
216	A_33_P3264667	0.006	2.001	CYP26C1	Homo sapiens cytochrome P450, family 26, subfamily C, polypeptide 1 (CYP26C1), mRNA [NM_183374]	NM_183374

The probes that represent the genes differentially up-regulated in macrophages cultured with iRBCs compared with those in macrophages cultured with RBCs were determined by using a moderated T-test. The application of the moderated T-test (P < 0.05, fold change > 2.0) led us to identify 310 probes that represent 216 annotated genes in the macrophages co-cultured with iRBCs.

[a]Fold change in upregulation indicates the mean expression level of the gene in macrophages co-cultured with iRBCs normalized to that in macrophages co-cultured with RBCs.

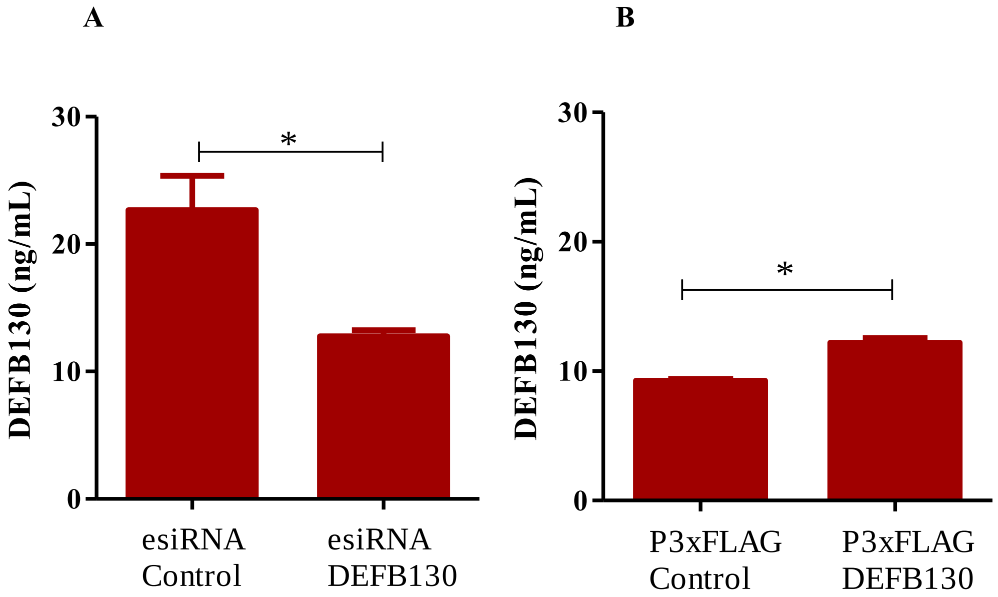

Supplementary Figure S1. Protein level of DEFB130 in transfected cell cultures. Phagocytic cells were incubated with enriched iRBCs (1:20) for 2h in 96-well plates. * indicates a significant difference between the groups as analyzed by Student's t test. A) Left panel for transfected primary macrophages with either control esiRNA or DEFB130-esiRNA. B) Right panel for transfected THP1 cells with ether empty vector plasmid P3XFLAG-CMV14 or P3XFLAG-CMV14-DEFB130.

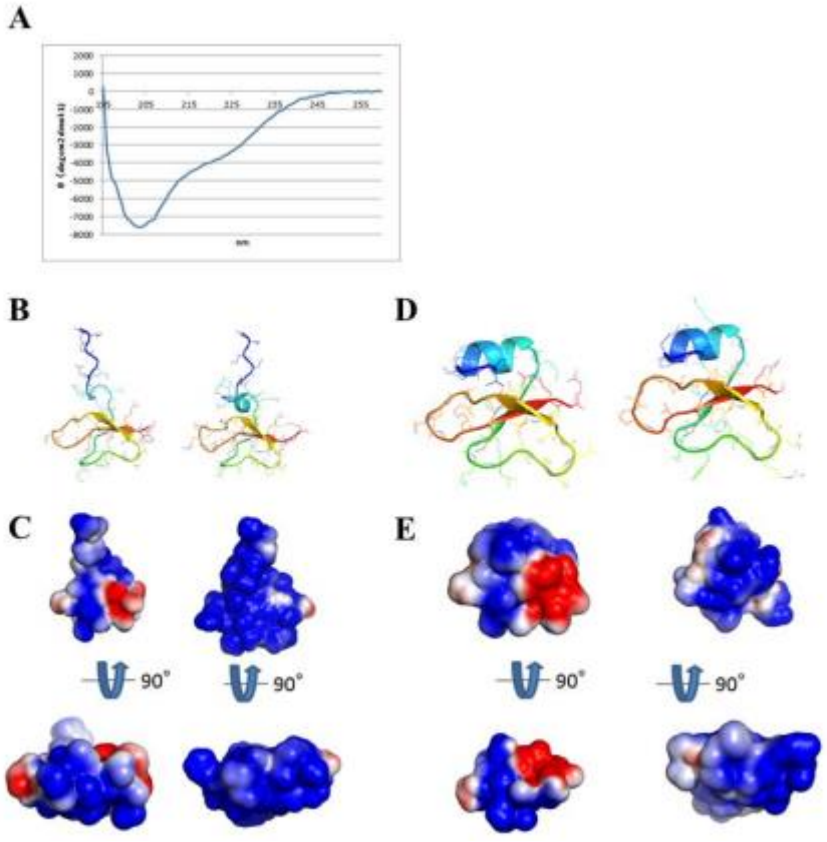

Supplementary Figure S2. Structure analysis of DEFB130. A) CD spectrum of recombinant DEFB130. B) A modelling structure of DEFB130 based on human DEFB3 (1KJ6). C) Electrostatic surface potentials of DEFB130 and DEFB3. The color of the surface potentials in the scale ranges from negatively charged (−1.5 kbT/ec; red) to positively charged amino acids (1.5 kbT/ec; blue). Left panel, DEFB130; right panel, DEFB3. D) A modelling structure of DEFB130 based on human DEFB2 (1FD3). E) Electrostatic surface potentials of DEFB130 and DEFB2. Left panel, DEFB130; right panel, DEFB2.

Supplementary Table S3. Primers used in this study for qRT-PCR.

Gene	Forward	Reverse
B-actin	CCTCACCCTGAAGTACCCCA	TCGTCCCAGTTGGTGACGAT
SMTNL2	AGCTGGCTTTCACCATGGC	CGATGAGGCGCTCACAGTT
DEFB130	ACTGGCGTTATTCCAGGACAA	GCACACCCCTTTCAAAGCAAT
RIT2	AAGCACCTGACACGGGTG	GAGGGACCGGAGGAAAAAAA
SYTL4	CTTCTGCCTGACAAGTCCCG	CCGCTTGATGCTGGTTTTTC
PRSS41	ATGCGTACATCCAGCCCATT	GCACGAAGTTGAAGGTGGAAG
GRP	AAACCACCAGCCACCTCAAC	CCACGAAGGCTGCTGATTG
ADAM19	TGACAGCAAGGGCCAACAC	GGCGGCTTGAGATGTTCAGA
